# Calcium oscillations optimize the energetic efficiency of mitochondrial metabolism

**DOI:** 10.1016/j.isci.2024.109078

**Published:** 2024-02-01

**Authors:** Valérie Voorsluijs, Francesco Avanzini, Gianmaria Falasco, Massimiliano Esposito, Alexander Skupin

**Affiliations:** 1 Luxembourg Centre for Systems Biomedicine, University of Luxembourg, 6 avenue du Swing, 4367 Belvaux, Luxembourg; 2 Complex Systems and Statistical Mechanics, Department of Physics and Materials Science, University of Luxembourg, 162 A avenue de la Faïencerie, 1511 Luxembourg, Luxembourg; 3 Department of Chemical Sciences, University of Padova, 1 Via F. Marzolo, 35131 Padova, Italy; 4 Department of Physics and Astronomy, University of Padova, 8 Via F. Marzolo, 35131 Padova, Italy; 5 Department of Physics and Materials Science, University of Luxembourg, 162 A avenue de la Faïencerie, 1511 Luxembourg, Luxembourg; 6 Department of Neuroscience, University of California, San Diego, 9500 Gilman Drive, San Diego, CA 92093, USA

**Keywords:** Natural sciences, Biological sciences, Human metabolism, Molecular biology

## Abstract

Energy transduction is central to living organisms, but the impact of enzyme regulation and signaling on its thermodynamic efficiency is generally overlooked. Here, we analyze the efficiency of ATP production by the tricarboxylic acid cycle and oxidative phosphorylation, which generate most of the chemical energy in eukaryotes. Calcium signaling regulates this pathway and can affect its energetic output, but the concrete energetic impact of this cross-talk remains elusive. Calcium enhances ATP production by activating key enzymes of the tricarboxylic acid cycle while calcium homeostasis is ATP-dependent. We propose a detailed kinetic model describing the calcium-mitochondria cross-talk and analyze it using nonequilibrium thermodynamics: after identifying the effective reactions driving mitochondrial metabolism out of equilibrium, we quantify the mitochondrial thermodynamic efficiency for different conditions. Calcium oscillations, triggered by extracellular stimulation or energy deficiency, boost the thermodynamic efficiency of mitochondrial metabolism, suggesting a compensatory role of calcium signaling in mitochondrial bioenergetics.

## Highlights

• A thermodynamically consistent model of Ca^2+^-mitochondria cross-talk is established
• The efficiency of Ca^2+^-regulated mitochondrial ATP synthesis is quantified
• Induction of Ca^2+^ spikes by energy deficiency or stimulation increases efficiency
• Oscillatory and not stationary Ca^2+^ concentration boosts mitochondrial efficiency

## Graphical abstract

**Figure:**
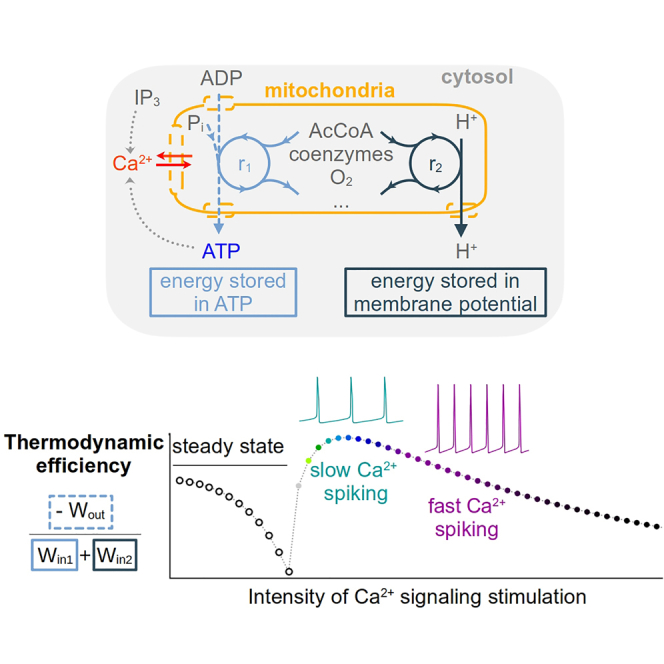

## Introduction

Life relies on permanent conversions between different forms of energy, a phenomenon referred to as energy transduction. A wide range of cellular processes are fueled by the chemical energy stored in adenosine triphosphate (ATP), but the compartmentalization of eukaryotic cells also enables the storage of potential energy across the membranes of organelles.^1^ Energy transduction is mediated by enzymes and pumps driven in a nonequilibrium thermodynamic manner by the hydrolysis of ATP, chemical gradients or membrane potentials.

In optimal scenarios where transduction is fully efficient, the input energy is completely transformed into usable work. However, biological processes are typically accompanied by entropy production, i.e., dissipation of energy in the form of heat and/or chemical waste that is unusable for transduction.^2^ For example, the action of many transmembrane ionic pumps transporting ions against their concentration gradient is often based on catalyzing the hydrolysis of ATP. The chemical energy released by hydrolysis is partly used to drive ionic transport while another part is dissipated. In the extreme case of pump uncoupling, also known as “slippage”, all the energy of ATP hydrolysis is dissipated without any ion transport.^3^

Different nonequilibrium kinetic models have been developed to account for energy loss in pumps^4^^,^^5^^,^^6^^,^^7^ but have only provided limited insights into energetic costs at the pathway level. New approaches based on metabolic network reconstruction and nonequilibrium thermodynamics are gradually emerging to rationalize the energetic costs of cellular processes^8^ including gene regulation,^9^ repair mechanisms,^10^^,^^11^ enzymatic catalysis,^12^ information processing^13^ or signaling.^14^^,^^15^ A framework to study energy transduction in complex open chemical reaction networks (CRN) has recently been proposed and used to study the efficiency of pathways of the central energy metabolism in the absence of regulations.^16^ Evaluating the efficiency of tightly coupled transduction processes, i.e., processes whose input and output currents are equal, is straightforward as it does not depend on the net reaction flux. However, when regulations come into play, this tight coupling can be lost and kinetic models become indispensable to evaluate the flux of the different processes contributing to the efficiency.

Here, we resort to such a kinetically-detailed nonequilibrium thermodynamic approach to show and quantify how active signaling can have a beneficial energetic impact on metabolism. In particular, we analyze the efficiency of the mitochondrial production of ATP via the tricarboxylic acid (TCA) cycle and oxidative phosphorylation (OXPHOS), and take into account its regulation by calcium (Ca^2+^). Ca^2+^ is a ubiquitous and versatile secondary messenger involved in processes ranging from energy metabolism to fertilization, muscle contraction, cell division or vesicle release.^17^^,^^18^ The binding of an extracellular agonist to a G protein-coupled receptor (GPCR) is one of the major triggers of cytosolic Ca^2+^ elevations. Depending on the cell type, typical agonists include neurotransmitters, hormones or metabolites and some GPCRs can thus work as sensors for the energetic cellular state, which can be relevant in the context of energy metabolism. Upon agonist binding, the activated G protein activates phospholipase C, which then cleaves phosphatidylinositol 4,5-bisphosphate (PIP_2_) into inositol 1,4,5-trisphosphate (IP_3_) and diacylglycerol. IP_3_ then binds to IP_3_ receptors (IP_3_Rs) located in the membrane of the endoplasmic reticulum (ER) and triggers Ca^2+^ release from this important intracellular Ca^2+^ reservoir. IP_3_Rs are also subjected to biphasic regulation by Ca^2+^. Indeed, at low concentrations, small Ca^2+^ release activates IP_3_Rs, which then tend to open more frequently. The resulting positive feedback loop is usually referred to as Ca^2+^-induced Ca^2+^ release (CICR). At high concentrations, however, Ca^2+^ binds to the inhibitory site of IP_3_Rs which then remain closed. This nonlinear release and its interplay with ER uptake can lead to oscillations in Ca^2+^ concentration of relatively large amplitude (about 1 μM) and occurring on a timescale of a few minutes to a few hours. Exchanges with non-ER compartments such as mitochondria or the extracellular space contribute to the fine-tuning of oscillations.^19^ More generally, sequestration mechanisms and oscillatory signaling prevent Ca^2+^ accumulation in the cytosol, as a persistent high cytosolic Ca^2+^ concentration is toxic for the cell.

Ca^2+^ is at the same time an important messenger for cell signaling and a regulator of energy metabolism. Due to Ca^2+^ exchanges, cytosolic Ca^2+^ oscillations are readily transmitted to mitochondria, where the regulation of energy metabolism occurs. Our kinetic model accounts for the allosteric regulation of the TCA cycle enzymes and for the regulation of OXPHOS by the mitochondrial membrane potential. In mitochondria, Ca^2+^ activates two key enzymes of the TCA cycle (isocitrate dehydrogenase and α-ketoglutarate dehydrogenase)^20^^,^^21^^,^^22^^,^^23^ and thereby increases the flux of high energy electrons, in the form of NADH, feeding the electron transport chain. The successive redox reactions in the mitochondrial membrane contribute to the establishment of the proton motive force driving the mitochondrial synthesis of ATP by F1F0-ATPase. Ca^2+^ influx in mitochondria can also slow down ATP synthesis by inducing a decrease in the mitochondrial membrane potential (ΔΨ), which reduces the proton motive force. Ca^2+^ thus stands out as an important regulator of mitochondrial energy metabolism.

**Figure 1:**
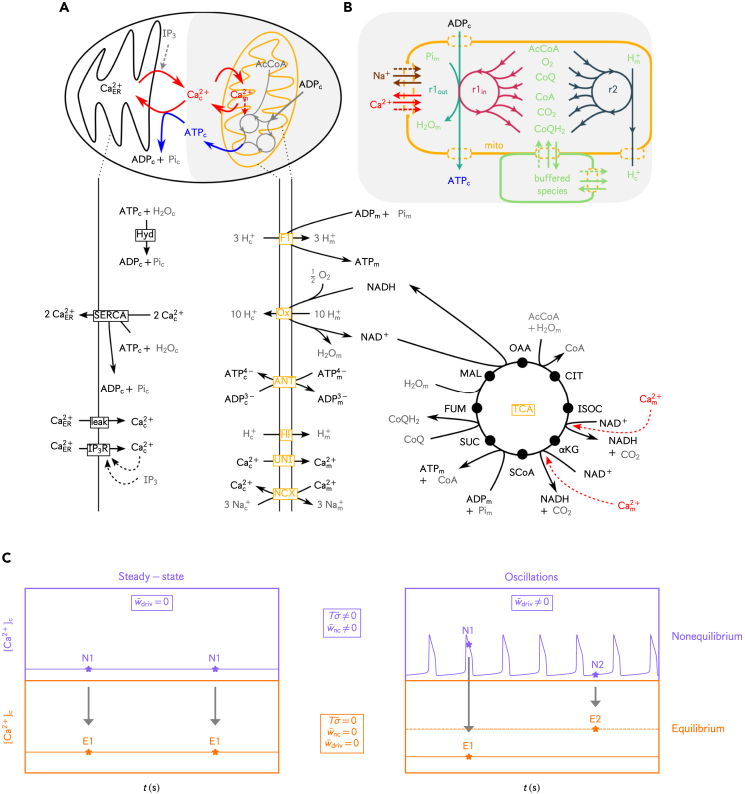
Representation of the model components, conceptualization of mitochondria as a chemical engine, expected work contributions to the thermodynamic efficiency in equilibrium and nonequilibrium conditions and corresponding abbreviations Balanced chemical equations, detailed expressions of the reaction rates, thermodynamic forces and reference parameter values are given in Tables 1, 2, 3, and 4, respectively. (A) The upper part depicts the Ca^2+^ (red) and ATP (blue) fluxes responsible for the cross-talk between Ca^2+^ dynamics and mitochondrial metabolism. The bottom part is a detailed description of the model components. The kinetic rates for TCA cycle fluxes and processes involving exchanges across the mitochondrial membrane are respectively originating from Dudycha^24^ and Magnus-Keizer models,^24−26.^ except for the transformation of MAL into OAA, which is described more realistically by a reversible flux.^25^ Here, OXPHOS corresponds to the net redox reaction resulting from the electron transport chain (Ox) and the synthesis of ATP by the F1F0-ATPase (F1). A last module, consisting of Ca^2+^ exchanges across the ER membrane and cytosolic ATP hydrolysis are taken from the models from Komin et al.^26^ and from Wacquier et al.^27^ Controlled species (i.e., species whose concentration is assumed to be constant) are shown in grey, dynamical species in black and dashed arrows represent regulations. Processes are annotated in yellow and black boxes for mitochondrial and cytosolic/ER processes, respectively. (B) Mitochondrial metabolism is conceptualized as an open chemical engine that transforms ADP_c_ into ATP_c_ through a set of 2 emergent cycles split in 3 effective reactions ($r1_{\text{out}}$, $r1_{\text{in}}$ and *r2*). Some of the controlled species involved in the internal reactions are buffered at a constant concentrations (green), while Na^+^ (brown) and Ca^2+^ (red) regulate reaction rates by activating specific enzymes or acting on the mitochondrial membrane potential. (C) Expected work contributions in equilibrium and nonequilibrium conditions. Stars denote the state of the system at different time points in nonequilibrium conditions (violet) and the corresponding underlying equilibrium state (orange). As illustrated, the non-zero driving work contribution in the oscillatory regime can modify the underlying equilibrium state of the system. Abbreviations: AcCoA – acetyl coenzyme A, αKG – alpha-ketoglutarate, ATP – Adenosine triphosphate, ADP – Adenosine diphosphate, CIT – citrate, CoA – coenzyme A, CoQ/CoQH_2_ – coenzyme Q10, FUM – fumarate, IP_3_ – inositol 1,4,5-trisphosphate, ISOC – isocitrate, MAL – malate, NAD^+^/NADH – nicotinamide adenine dinucleotide, OAA – oxaloacetate, Pi – inorganic phosphate, SUC – succinate, SCoA – succinyl coenzyme A.

Contrarily to mitochondrial uptake, Ca^2+^ sequestration into the ER via the sarcoendoplasmic reticulum Ca^2+^ ATPase (SERCA) or extrusion to the extracellular space via the plasma membrane Ca^2+^ ATPase (PMCA) are ATP-dependent. If we focus on the intracellular exchanges, the central coupling enabling the Ca^2+^-mitochondria cross-talk is thus given by the Ca^2+^ fluxes between the cytosol and the ER or mitochondria (Figure 1A), where Ca^2+^ release from the ER, by leakage or via channels (IP_3_Rs) upon stimulation by IP_3_, and Ca^2+^ exchanges with mitochondria are ATP-independent, as opposed to Ca^2+^ transport into the ER that relies on ATP-consuming SERCA pumps.

Since intracellular Ca^2+^ dynamics is strongly nonlinear, impacts mitochondrial ATP synthesis and depends itself on ATP availability, evaluating the net effect of signaling on the energetic efficiency of mitochondrial metabolism is not straightforward. Our analysis quantifies the energetic efficiency of this essential cellular process beyond steady-state conditions, such as in an oscillatory regime. Notably, the dual impact of Ca^2+^ signaling on ATP level exists for other signaling molecules and regulators (e.g., the regulation of energy metabolism by AMPK signaling) and the nontrivial effects we report here could thus be relevant for other pathways. Overall, the proposed framework is laying the foundations for a more comprehensive characterization of energetic costs in biology.

## Results

### Modeling and theoretical frameworks

We developed a curated model for the essential Ca^2+^-metabolism system by integrating different modules.^24^^,^^25^^,^^26^^,^^27^^,^^30^^,^^31^^,^^32^^,^^33^^,^^34^^,^^35^ Balanced chemical equations, detailed expressions of the reaction rates, thermodynamic forces and reference parameter values are given in Tables 1, 2, 3, and 4, respectively. The model was calibrated to reproduce key features of the Ca^2+^-metabolism cross-talk based on experimental data from cultured astrocytes,^36^ to generate physiological concentrations and concentration ratios with a realistic mitochondrial membrane potential (Figures S1 and S2 and Table 5), and to be thermodynamically consistent (details in Model calibration in the STAR Methods). The interplay between Ca^2+^ oscillations and mitochondrial energy metabolism has been at the core of long-standing experimental and computational investigations and is well-characterized from a kinetic point of view.^20^^,^^21^^,^^22^^,^^23^ The underlying kinetic models originally aimed at capturing the essential mechanisms of the Ca^2+^-metabolism interplay and at rationalizing experimental data about the response of Ca^2+^ signals to changes in mitochondrial activity (and vice versa). After refining these models to combine them in a coherent way (details section kinetic model of the STAR Methods), we analyzed the coupled pathways by using a nonequilibrium thermodynamic description of CRN.^16^^,^^37^^,^^38^^,^^39^^,^^40^^,^^41^^,^^42^^,^^43^

**Table 1:** Chemical reactions incorporated into the kinetic model

**Calcium exchanges and cytosolic ATP dynamics**		

ERout	IP_3_ receptors and leakage by passive diffusion from the ER	${\text{Ca}}_{\text{ER}}^{2+}\overset{{\text{IP}}_{3}}{⇌}{\text{Ca}}_{c}^{2+}$
SERCA	Sarco/endoplasmic reticulum Ca^2+^ ATPase	$2\;{\text{Ca}}_{c}^{2+}+{\text{ATP}}_{c}+H_{2}O_{c}⇌2\;{\text{Ca}}_{\text{ER}}^{2+}+{\text{ADP}}_{c}+{\text{Pi}}_{c}$
NCX	Mitochondrial Na^+^/Ca^2+^ exchanger	${\text{Ca}}_{m}^{2+}+3\;{\text{Na}}_{c}^{+}⇌{\text{Ca}}_{c}^{2+}+3\;{\text{Na}}_{m}^{+}$
UNI	Mitochondrial uniporter	${\text{Ca}}_{c}^{2+}⇌{\text{Ca}}_{m}^{2+}$
Hyd	Cytosolic ATP hydrolysis due to cellular activity	${\text{ATP}}_{c}+H_{2}O_{c}⇌{\text{ADP}}_{c}+{\text{Pi}}_{c}$

**Electron transport chain and oxidative phosphorylation**		

OX	Cellular respiration	$\text{NADH}+10\;H_{m}^{+}+\frac{1}{2}O_{2}⇌\text{NAD}+10\;H_{c}^{+}+H_{2}O_{m}$
F1	F1F0-ATPase	${\text{ADP}}_{m}+{\text{Pi}}_{m}+3\;H_{c}^{+}⇌{\text{ATP}}_{m}+H_{2}O_{m}+3\;H_{m}^{+}$

**TCA cycle**		

CS	Citrate synthase	$\text{OAA}+\text{AcCoA}+H_{2}O_{m}⇌\text{CIT}+\text{CoA}$
ACO	Aconitase	CIT⇌ISOC
IDH	Isocitrate dehydrogenase	$\text{ISOC}+\text{NAD}⇌\alpha \text{KG}+\text{NADH}+{\text{CO}}_{2}$
KGDH	α-ketoglutarate dehydrogenase	$\alpha \text{KG}+\text{NAD}+\text{CoA}⇌\text{SCoA}+\text{NADH}+{\text{CO}}_{2}$
SL	Succinyl-CoA Synthetase	$\text{SCoA}+{\text{ADP}}_{m}+{\text{Pi}}_{m}⇌\text{SUC}+{\text{ATP}}_{m}+\text{CoA}$
SDH	Succinate dehydrogenase	$\text{SUC}+\text{CoQ}⇌\text{FUM}+{\text{CoQH}}_{2}$
FH	Fumarate hydratase	$\text{FUM}+H_{2}O_{m}⇌\text{MAL}$
MDH	Malate dehydrogenase	MAL+NAD⇌OAA+NADH

**Other exchange processes**		

ANT	Adenine nucleotide transporter	${\text{ATP}}_{m}+{\text{ADP}}_{c}⇌{\text{ATP}}_{c}+{\text{ADP}}_{m}$
Hl	Mitochondrial proton leak	$H_{c}^{+}⇌H_{m}^{+}$

Subscripts c, ER and m refer to the cytosol, the endoplasmic reticulum and the mitochondria, respectively.

**Table 2:** Fluxes of the system

Process	$V_{\text{ref}}$	$J_{\kappa }\left({\text{mMs}}^{-1}\right)$	Reference
ACO	$V_{m}$	$J_{\text{ACO}}=k_{f}^{\text{ACO}}\left({\left[\text{CIT}\right]}_{m}-\frac{{\left[\text{ISOC}\right]}_{m}}{K_{\text{ACO}}}\right)$	Cortassa et al.^33^
ANT	$V_{m}$	$J_{\text{ANT}}=V_{\max}^{\text{ANT}}\frac{1-\frac{{\left[{\text{ATP}}^{4-}\right]}_{c}{\left[{\text{ADP}}^{3-}\right]}_{m}}{{\left[{\text{ATP}}^{4-}\right]}_{m}{\left[{\text{ADP}}^{3-}\right]}_{c}}e^{\frac{-F\Delta \Psi }{RT}}}{\left(1+\frac{{\left[{\text{ATP}}^{4-}\right]}_{c}}{{\left[{\text{ADP}}^{3-}\right]}_{c}}e^{\frac{-fF\Delta \Psi }{RT}}\right)\left(1+\frac{{\left[{\text{ADP}}^{3-}\right]}_{m}}{{\left[{\text{ATP}}^{4-}\right]}_{m}}\right)}$	Magnus and Keizer^30^
CS	$V_{m}$	$J_{\text{CS}}=\frac{V_{\max}^{\text{CS}}}{1+\frac{K_{M,\text{AcCoA}}}{{\left[\text{AcCoA}\right]}_{m}}+\frac{K_{M,\text{OAA}}^{\text{CS}}}{{\left[\text{OAA}\right]}_{m}}\left(1+\frac{{\left[\text{AcCoA}\right]}_{m}}{K_{i,\text{AcCoA}}}\right)+\frac{K_{s,\text{AcCoA}}K_{M,\text{OAA}}^{\text{CS}}}{{\left[\text{OAA}\right]}_{m}{\left[\text{AcCoA}\right]}_{m}}}$	Dudycha^24^
ERout	$V_{c}$	$J_{\text{ERout}}=\left(V_{\max}^{{\text{IP}}_{3}R}\frac{{\left[{\text{IP}}_{3}\right]}^{2}}{{\left[{\text{IP}}_{3}\right]}^{2}+K_{a,{\text{IP}}_{3}}^{2}}\frac{{\left[{\text{Ca}}^{2+}\right]}_{c}^{2}}{{\left[{\text{Ca}}^{2+}\right]}_{c}^{2}+K_{a,\text{Cac}}^{2}}\frac{K_{i,\text{Ca}}^{4}}{K_{i,\text{Ca}}^{4}+{\left[{\text{Ca}}^{2+}\right]}_{c}^{4}}+V^{\text{LEAK}}\right)\left({\left[{\text{Ca}}^{2+}\right]}_{\text{ER}}-{\left[{\text{Ca}}^{2+}\right]}_{c}\right)$	Komin et al.^26^
F1	$V_{m}$	$J_{F1}=-\rho_{f1}\frac{\left[p_{a}10^{3\Delta \text{pH}}+p_{c1}e^{\frac{3F\Delta \Psi_{B}}{RT}}\right]A_{F1}-p_{a}e^{\frac{3F\Delta \Psi }{RT}}+p_{c2}A_{F1}e^{\frac{3F\Delta \Psi }{RT}}}{\left[1+p_{1}A_{F1}\right]e^{\frac{3F\Delta \Psi_{B}}{RT}}+\left[p_{2}+p_{3}A_{F1}\right]e^{\frac{3F\Delta \Psi }{RT}}}$	Magnus and Keizer^30^
		with $A_{F1}=K_{F1}\frac{{\left[\text{ATP}\right]}_{m}}{{\left[\text{ADP}\right]}_{m}{\left[\text{Pi}\right]}_{m}}$	
FH	$V_{m}$	$J_{\text{FH}}=k_{f}^{\text{FH}}\left({\left[\text{FUM}\right]}_{m}-\frac{{\left[\text{MAL}\right]}_{m}}{K_{\text{FH}}}\right)$	Cortassa et al.^33^
Hl	$V_{m}$	$J_{\text{Hl}}=g_{H}\left(\Delta \Psi -2.303\frac{RT}{F}\Delta \text{pH}\right)$	Magnus and Keizer^30^
Hyd	$V_{c}$	$J_{\text{Hyd}}=k_{\text{Hyd}}\frac{{\left[\text{ATP}\right]}_{c}}{{\left[\text{ATP}\right]}_{c}+K_{M,\text{ATPc}}}$	Wacquier et al.^27^
IDH	$V_{m}$	$J_{\text{IDH}}=\frac{V_{\max}^{\text{IDH}}}{1+\frac{{\left[H\right]}_{m}}{k_{h,1}}+\frac{k_{h,2}}{{\left[H\right]}_{m}}+\frac{{\left(\frac{K_{M,\text{ISOC}}}{{\left[\text{ISOC}\right]}_{m}}\right)}^{n_{i}}}{\left(1+\frac{{\left[\text{ADP}\right]}_{m}}{K_{a,\text{ADP}}}\right)\left(1+\frac{{\left[{\text{Ca}}^{2+}\right]}_{m}}{K_{a,\text{Cam}}}\right)}+\frac{K_{M,\text{NAD}}^{\text{IDH}}}{{\left[\text{NAD}\right]}_{m}}\left(1+\frac{{\left[\text{NADH}\right]}_{m}}{K_{i,\text{NADH}}}\right)+\frac{{\left(\frac{K_{M,\text{ISOC}}}{{\left[\text{ISOC}\right]}_{m}}\right)}^{n_{i}}\frac{K_{M,\text{NAD}}^{\text{IDH}}}{{\left[\text{NAD}\right]}_{m}}\left(1+\frac{{\left[\text{NADH}\right]}_{m}}{K_{i,\text{NADH}}}\right)}{\left(1+\frac{{\left[\text{ADP}\right]}_{m}}{K_{a,\text{ADP}}}\right)\left(1+\frac{{\left[{\text{Ca}}^{2+}\right]}_{m}}{K_{a,\text{Cam}}}\right)}}$	Cortassa et al.^33^
KGDH	$V_{m}$	$J_{\text{KGDH}}=\frac{V_{\max}^{\text{KGDH}}}{1+\frac{\frac{K_{M,\alpha \text{KG}}}{{\left[\alpha \text{KG}\right]}_{m}}{\left(\frac{K_{M,\text{NAD}}^{\text{KGDH}}}{{\left[\text{NAD}\right]}_{m}}\right)}^{n_{\alpha \text{KG}}}}{\left(1+\frac{{\left[{\text{Mg}}^{2+}\right]}_{m}}{K_{D,\text{Mg}}}\right)\left(1+\frac{{\left[{\text{Ca}}^{2+}\right]}_{m}}{K_{D,\text{Ca}}}\right)}}$	Dudycha^24^
MDH	$V_{m}$	$J_{\text{MDH}}=V_{\max}^{\text{MDH}}\frac{{\left[\text{MAL}\right]}_{m}{\left[\text{NAD}\right]}_{m}-\frac{{\left[\text{OAA}\right]}_{m}{\left[\text{NADH}\right]}_{m}}{K_{\text{MDH}}}}{\left(1+\frac{{\left[\text{MAL}\right]}_{m}}{K_{M,\text{MAL}}}\right)\left(1+\frac{{\left[\text{NAD}\right]}_{m}}{K_{M,\text{NAD}}^{\text{MDH}}}\right)+\left(1+\frac{{\left[\text{OAA}\right]}_{m}}{K_{M,\text{OAA}}^{\text{MDH}}}\right)\left(1+\frac{{\left[\text{NADH}\right]}_{m}}{K_{M,\text{NADH}}}\right)-1}$	Berndt et al.^25^
NCX	$V_{m}$	$J_{\text{NCX}}=V_{\max}^{\text{NCX}}\frac{e^{\frac{bF\left(\Delta \Psi -\Delta \Psi^{*}\right)}{RT}}}{{\left(1+\frac{K_{M,\text{Na}}}{{\left[{\text{Na}}^{+}\right]}_{c}}\right)}^{n}\left(1+\frac{K_{M,\text{Ca}}}{{\left[{\text{Ca}}^{2+}\right]}_{m}}\right)}\frac{{\left[{\text{Ca}}^{2+}\right]}_{m}}{{\left[{\text{Ca}}^{2+}\right]}_{c}}$	Cortassa et al.^33^
Ox	$V_{m}$	$J_{\text{Ox}}=\frac{1}{2}\rho_{\text{res}}\frac{\left[r_{a}10^{6\Delta \text{pH}}+r_{c1}e^{\frac{6F\Delta \Psi_{B}}{RT}}\right]A_{\text{res}}-r_{a}e^{\frac{g6F\Delta \Psi }{RT}}+r_{c2}A_{\text{res}}e^{\frac{g6F\Delta \Psi }{RT}}}{\left[1+r_{1}A_{\text{res}}\right]e^{\frac{6F\Delta \Psi_{B}}{RT}}+\left[r_{2}+r_{3}A_{\text{res}}\right]e^{\frac{g6F\Delta \Psi }{RT}}}$	Magnus and Keizer^30^
		with $A_{\text{res}}=K_{\text{res}}\sqrt{\frac{{\left[\text{NADH}\right]}_{m}}{{\left[\text{NAD}\right]}_{m}}}$	
SDH	$V_{m}$	$J_{\text{SDH}}=\frac{V_{\max}^{\text{SDH}}}{1+\frac{K_{M,\text{SUC}}}{{\left[\text{SUC}\right]}_{m}}\left(1+\frac{{\left[\text{OAA}\right]}_{m}}{K_{i,\text{OAA}}}\right)\left(1+\frac{{\left[\text{FUM}\right]}_{m}}{K_{i,\text{FUM}}}\right)}$	Cortassa et al.^33^
SERCA	$V_{c}$	$J_{\text{SERCA}}=V_{\max}^{\text{SERCA}}\frac{{\left[{\text{Ca}}^{2+}\right]}_{c}^{2}}{{\left[{\text{Ca}}^{2+}\right]}_{c}^{2}+K_{\text{Ca}}^{2}}\frac{{\left[\text{ATP}\right]}_{c}}{{\left[\text{ATP}\right]}_{c}+K_{\text{ATPc}}}$	Wacquier et al.^27^
SL	$V_{m}$	$J_{\text{SL}}=k_{f}^{\text{SL}}\left({\left[\text{SCoA}\right]}_{m}{\left[\text{ADP}\right]}_{m}{\left[\text{Pi}\right]}_{m}-\frac{{\left[\text{SUC}\right]}_{m}{\left[\text{ATP}\right]}_{m}{\left[\text{CoA}\right]}_{m}}{K_{\text{SL}}}\right)$	Wei et al.^35^
UNI	$V_{m}$	$J_{\text{UNI}}=V_{\max}^{\text{UNI}}\frac{2F\left(\Delta \Psi -\Delta \Psi^{*}\right)}{RT\left(1-e^{-\frac{2F\left(\Delta \Psi -\Delta \Psi^{*}\right)}{RT}}\right)}\frac{\frac{{\left[{\text{Ca}}^{2+}\right]}_{c}}{K_{\text{trans}}}{\left(1+\frac{{\left[{\text{Ca}}^{2+}\right]}_{c}}{K_{\text{trans}}}\right)}^{3}}{{\left(1+\frac{{\left[{\text{Ca}}^{2+}\right]}_{c}}{K_{\text{trans}}}\right)}^{4}+\frac{L}{{\left(1+\frac{{\left[{\text{Ca}}^{2+}\right]}_{c}}{K_{\text{act}}}\right)}^{n_{a}}}}$	Magnus and Keizer^30^

$V_{\text{ref}}$ is the volume of reference with respect to which each reaction rate, $J_{\kappa }$, and the corresponding entropy production rate, ${\dot{\sigma }}_{\kappa }$, are normalized. Starting from the corresponding pseudoisomer concentrations, Magnus and Keizer estimate that ${\left[{\text{ATP}}^{4-}\right]}_{c}=0.05\;{\left[\text{ATP}\right]}_{c}$, ${\left[{\text{ATP}}^{4-}\right]}_{m}=0.05\;{\left[\text{ATP}\right]}_{m}$, ${\left[{\text{ADP}}^{3-}\right]}_{c}=0.45\;{\left[\text{ADP}\right]}_{c}$ and ${\left[{\text{ADP}}^{3-}\right]}_{m}=0.36\;{\left[\text{ADP}\right]}_{m}$.

**Table 3:** Forces of the system

Process	$\Delta_{r}G_{\kappa }^{\prime }\left(J\;{\text{mol}}^{-1}\right)$	
ACO	$\Delta_{r}G_{\text{ACO},m}^{\prime }=\Delta_{r}G_{\text{ACO},m}^{\prime \circ }+RT\;\ln\frac{{\left[\text{ISOC}\right]}_{m}}{{\left[\text{CIT}\right]}_{m}}$	$\Delta_{r}G_{\text{ACO},m}^{\prime \circ }=6700\;J\;{\text{mol}}^{-1}$
ANT	$\Delta_{r}G_{\text{ANT},m}^{\prime }=RT\;\ln\frac{{\left[{\text{ATP}}^{4-}\right]}_{c}{\left[{\text{ADP}}^{3-}\right]}_{m}}{{\left[{\text{ATP}}^{4-}\right]}_{m}{\left[{\text{ADP}}^{3-}\right]}_{c}}-F\Delta \Psi$	
CS	$\Delta_{r}G_{\text{CS},m}^{\prime }=\Delta_{r}G_{\text{CS},m}^{\prime \circ }+RT\;\ln\frac{{\left[\text{CIT}\right]}_{m}{\left[\text{CoA}\right]}_{m}}{{\left[\text{OAA}\right]}_{m}{\left[\text{AcCoA}\right]}_{m}}$	$\Delta_{r}G_{\text{CS},m}^{\prime \circ }=-41200\;J\;{\text{mol}}^{-1}$
ERout	$\Delta_{r}G_{\text{ERout},c}^{\prime }=RT\;\ln\frac{{\left[{\text{Ca}}^{2+}\right]}_{c}}{{\left[{\text{Ca}}^{2+}\right]}_{\text{ER}}}$	
F1	$\Delta_{r}G_{F1,m}^{\prime }=-\Delta_{r}G_{\text{Hyd},m}^{\prime \circ }+RT\;\ln\frac{{\left[H\right]}_{m}^{3}{\left[\text{ATP}\right]}_{m}}{{\left[H\right]}_{c}^{3}{\left[\text{ADP}\right]}_{m}{\left[\text{Pi}\right]}_{m}}-3F\Delta \Psi$	$\Delta_{r}G_{\text{Hyd},m}^{\prime \circ }=-32200\;J\;{\text{mol}}^{-1}$
FH	$\Delta_{r}G_{\text{FH},m}^{\prime }=\Delta_{r}G_{\text{FH},m}^{\prime \circ }+RT\;\ln\frac{{\left[\text{MAL}\right]}_{m}}{{\left[\text{FUM}\right]}_{m}}$	$\Delta_{r}G_{\text{FH},m}^{\prime \circ }=-3400\;J\;{\text{mol}}^{-1}$
Hl	$\Delta_{r}G_{\text{Hl},m}^{\prime }=RT\;\ln\frac{{\left[H\right]}_{m}}{{\left[H\right]}_{c}}-F\Delta \Psi$	
Hyd	$\Delta_{r}G_{\text{Hyd},c}^{\prime }=\Delta_{r}G_{\text{Hyd},c}^{\prime \circ }+RT\;\ln\frac{{\left[\text{ADP}\right]}_{c}{\left[\text{Pi}\right]}_{c}}{{\left[\text{ATP}\right]}_{c}}$	$\Delta_{r}G_{\text{Hyd},c}^{\prime \circ }=-28300\;J\;{\text{mol}}^{-1}$
IDH	$\Delta_{r}G_{\text{IDH},m}^{\prime }=\Delta_{r}G_{\text{IDH},m}^{\prime \circ }+RT\;\ln\frac{{\left[\alpha \text{KG}\right]}_{m}{\left[{\text{CO}}_{2}\right]}_{m}{\left[\text{NADH}\right]}_{m}}{{\left[\text{ISOC}\right]}_{m}{\left[\text{NAD}\right]}_{m}}$	$\Delta_{r}G_{\text{IDH},m}^{\prime \circ }=5100\;J\;{\text{mol}}^{-1}$
KGDH	$\Delta_{r}G_{\text{KGDH},m}^{\prime }=\Delta_{r}G_{\text{KGDH},m}^{\prime \circ }+RT\;\ln\frac{{\left[\text{SCoA}\right]}_{m}{\left[\text{NADH}\right]}_{m}{\left[{\text{CO}}_{2}\right]}_{m}}{{\left[\alpha \text{KG}\right]}_{m}{\left[\text{NAD}\right]}_{m}{\left[\text{CoA}\right]}_{m}}$	$\Delta_{r}G_{\text{KGDH},m}^{\prime \circ }=-27600\;J\;{\text{mol}}^{-1}$
MDH	$\Delta_{r}G_{\text{MDH},m}^{\prime }=\Delta_{r}G_{\text{MDH},m}^{\prime \circ }+RT\;\ln\frac{{\left[\text{OAA}\right]}_{m}{\left[\text{NADH}\right]}_{m}}{{\left[\text{NAD}\right]}_{m}{\left[\text{MAL}\right]}_{m}}$	$\Delta_{r}G_{\text{MDH},m}^{\prime \circ }=24200\;J\;{\text{mol}}^{-1}$
NCX	$\Delta_{r}G_{\text{NCX},m}^{\prime }=RT\;\ln\frac{{\left[{\text{Ca}}^{2+}\right]}_{c}{\left[\text{Na}\right]}_{m}^{3}}{{\left[{\text{Ca}}^{2+}\right]}_{m}{\left[\text{Na}\right]}_{c}^{3}}-F\Delta \Psi$	
Ox	$\Delta_{r}G_{\text{Ox},m}^{\prime }=\Delta_{r}G_{\text{Ox},m}^{\prime \circ }+RT\;\ln\frac{{\left[H\right]}_{c}^{10}{\left[\text{NAD}\right]}_{m}}{{\left[H\right]}_{m}^{10}{\left[\text{NADH}\right]}_{m}{\left[O_{2}\right]}_{m}^{0.5}}+10F\Delta \Psi$	$\Delta_{r}G_{\text{Ox},m}^{\prime \circ }=-225300\;J\;{\text{mol}}^{-1}$
SDH	$\Delta_{r}G_{\text{SDH},m}^{\prime }=\Delta_{r}G_{\text{SDH},m}^{\prime \circ }+RT\;\ln\frac{{\left[\text{FUM}\right]}_{m}{\left[{\text{CoQH}}_{2}\right]}_{m}}{{\left[\text{SUC}\right]}_{m}{\left[\text{CoQ}\right]}_{m}}$	$\Delta_{r}G_{\text{SDH},m}^{\prime \circ }=-24200\;J\;{\text{mol}}^{-1}$
SERCA	$\Delta_{r}G_{\text{SERCA},c}^{\prime }=\Delta_{r}G_{\text{Hyd},c}^{\prime \circ }+RT\;\ln\frac{{\left[\text{ADP}\right]}_{c}{\left[\text{Pi}\right]}_{c}{\left[{\text{Ca}}^{2+}\right]}_{\text{ER}}^{2}}{{\left[\text{ATP}\right]}_{c}{\left[{\text{Ca}}^{2+}\right]}_{c}^{2}}$	$\Delta_{r}G_{\text{Hyd},c}^{\prime \circ }=-28300\;J\;{\text{mol}}^{-1}$
SL	$\Delta_{r}G_{\text{SL},m}^{\prime }=\Delta_{r}G_{\text{SL},m}^{\prime \circ }+RT\;\ln\frac{{\left[\text{SUC}\right]}_{m}{\left[\text{CoA}\right]}_{m}{\left[\text{ATP}\right]}_{m}}{{\left[\text{SCoA}\right]}_{m}{\left[\text{ADP}\right]}_{m}{\left[\text{Pi}\right]}_{m}}$	$\Delta_{r}G_{\text{SL},m}^{\prime \circ }=800\;J\;{\text{mol}}^{-1}$
UNI	$\Delta_{r}G_{\text{UNI},m}^{\prime }=RT\;\ln\frac{{\left[{\text{Ca}}^{2+}\right]}_{m}}{{\left[{\text{Ca}}^{2+}\right]}_{c}}-2F\Delta \Psi$	

Transformed Gibbs free energies of reaction ($\Delta_{r}G_{\kappa }$) associated to each process of the system. The indices *c* and *m* associated to $\Delta_{r}G_{\kappa }^{\prime \circ }$ indicate that this thermodynamic quantity is evaluated at cytosolic and mitochondrial pH, that is, pH=7.2 and pH=8.0, respectively. The value of $\Delta_{r}G_{\kappa }^{\prime \circ }$, which also accounts for physiological ionic strength (I=0.12 M^28^) and ${\left[{\text{Mg}}^{2+}\right]}_{m}$ (pMg = 3.4), was retrieved for each relevant process via Equilibrator.^29^ Starting from the corresponding pseudoisomer concentrations, Magnus and Keizer estimate that ${\left[{\text{ATP}}^{4-}\right]}_{c}=0.05\;{\left[\text{ATP}\right]}_{c}$, ${\left[{\text{ATP}}^{4-}\right]}_{m}=0.05\;{\left[\text{ATP}\right]}_{m}$, ${\left[{\text{ADP}}^{3-}\right]}_{c}=0.45\;{\left[\text{ADP}\right]}_{c}$ and ${\left[{\text{ADP}}^{3-}\right]}_{m}=0.36\;{\left[\text{ADP}\right]}_{m}$.

**Table 4:** Reference parameter values

Parameter	Definition	Value (units)	Reference
α	Ratio between ER and cytosol volumes	0.10	Wacquier et al.^27^
$A_{\text{tot}}$	Total concentration of cytosolic adenine nucleotides	3 mM	Moein^36^
$A_{m,\text{tot}}$	Total concentration of mitochondrial adenine nucleotides	15 mM	Magnus and Keizer^30^
*b*	Dependence of electrogenic Na^+^/Ca^2+^ exchanger on ΔΨ	0.5	Magnus and Keizer^30^
$C_{m}$	Mitochondrial membrane capacitance	$1.812\times 10^{-3}$ mM mV^−1^	Cortassa et al.^33^
$\left[{\text{CO}}_{2}\right]$	Total CO_2_ concentration in mitochondrial matrix	21.4 mM	Wu et al.^56^
[CoA]	CoA concentration in mitochondrial matrix	0.02 mM	Cortassa et al.^33^
[CoQ]	CoQ concentration in mitochondrial matrix	0.97 mM	Wu et al.^56^
$\left[{\text{CoQH}}_{2}\right]$	CoQ_2_ concentration in mitochondrial matrix	0.38 mM	Wu et al.^56^
$c_{\text{tot}}$	Total free Ca^2+^ concentration of the cell normalized by $V_{c}$	1500 μM	This work
$c_{\text{Ktot}}$	Total concentration of TCA cycle intermediates	1 mM	Cortassa et al.^33^
δ	Ratio between mitochondrial matrix and cytosol volumes	0.15	Siess et al.^57^, Lund and Wiggins^58^
ΔpH	pH difference between cytosol and mitochondrial matrix (${\text{pH}}_{c}-{\text{pH}}_{m}$)	−0.80	Buckler and Vaughan-Jones^59^, Casey et al.^60^
$\Delta \Psi^{*}$	Membrane potential offset for Ca^2+^ transport	91 mV	Magnus and Keizer^30^
$\Delta \Psi_{B}$	Total phase boundary potential	50 mV	Magnus and Keizer^30^
*F*	Faraday constant	96.485 kC mol^−1^	
*f*	Fraction of ΔΨ responsible for the behavior of ANT in energized mitochondria	0.5	Magnus and Keizer^30^
$f_{c}$	Fraction of free cytosolic Ca^2+^	0.01	Wacquier et al.^27^
$f_{e}$	Fraction of free Ca^2+^ in the ER	0.01	Wacquier et al.^27^
$f_{m}$	Fraction of free mitochondrial Ca^2+^	0.0003	Magnus and Keizer^30^
γ	Conversion factor between mM and μM	1000 μM mM^−1^	
*g*	Fitting factor for voltage in respiration rate	0.85	Magnus and Keizer^30^
$g_{H}$	Ionic conductance of the mitochondrial inner membrane	$10^{-5}$ mM mV^−1^ s^−1^	Cortassa et al.^33^
${\left[H^{+}\right]}_{c}$	Cytosolic proton concentration	$6.31\times 10^{-5}$ mM	Buckler and Vaughan-Jones^59^, Casey et al.^60^
${\left[H^{+}\right]}_{m}$	Concentration of proton in the mitochondrial matrix	$10^{-5}$ mM	Buckler and Vaughan-Jones^59^, Casey et al.^60^
$K_{a,\text{Cac}}$	Activation constant of IP_3_Rs for cytosolic Ca^2+^	0.70 μM	This work
$K_{\text{ACO}}$	Equilibrium constant of ACO	0.067	Berndt et al.^25^, Flamholz et al.^29^
$K_{\text{act}}$	Dissociation constant of mitochondrial uniporter for activating Ca^2+^	0.38	Magnus and Keizer^30^
$K_{\text{ATPc}}$	Dissociation constant of SERCA for cytosolic ATP	0.05 mM	Moein^36^, Scofano et al.^61^
$K_{a,\text{ADP}}$	Activation constant of IDH for ADP_m_	0.062 mM	Dudycha^24^, Cortassa et al.^33^
$K_{a,\text{Cam}}$	Activation constant of IDH for mitochondrial Ca^2+^	1.41 μM	Cortassa et al.^33^
$K_{a,{\text{IP}}_{3}}$	Activation constant of IP_3_Rs for IP_3_	1.00 μM	Wacquier et al.^27^, Dupont and Erneux^62^
$K_{\text{Ca}}$	Dissociation constant of SERCA for Ca^2+^	0.35 μM	Wacquier et al.^27^, Dupont and Erneux^62^
$K_{D,\text{Ca}}$	Dissociation constant of KGDH for mitochondrial Ca^2+^	1.27 μM	Dudycha^24^, Cortassa et al.^33^
$K_{D,\text{Mg}}$	Dissociation constant of KGDH for mitochondrial Mg^2+^	0.0308 mM	Cortassa et al.^33^
$K_{F1}$	Equilibrium constant for ATP hydrolysis in mitochondrial matrix	$1.71\times 10^{6}$	Cortassa et al.^33^, Pietrobon and Caplan^63^
$K_{\text{FH}}$	Equilibrium constant for FH	3.942	Flamholz et al.^29^
$k_{f}^{\text{ACO}}$	Forward rate constant of ACO	12.5 s^−1^	Cortassa et al.^33^
$k_{f}^{\text{FH}}$	Forward rate constant of FH	8.3 s^−1^	This work
$k_{f}^{\text{SL}}$	Forward rate constant of SL	0.127 mM^−2^ s^−1^	Cortassa et al.^33^
$k_{h,1}$	First ionization constant of IDH	$8.1\times 10^{-5}$ mM	Dudycha^24^, Cortassa et al.^33^
$k_{h,2}$	Second ionization constant of IDH	$5.98\times 10^{-5}$ mM	Dudycha^24^, Cortassa et al.^33^
$k_{\text{Hyd}}$	Hydrolysis rate of ATP_c_ due to cellular activity	$9\times 10^{-2}$ mM s^−1^	This work
$K_{i,\text{AcCoA}}$	Inhibition constant of CS for AcCoA	$3.7068\times 10^{-2}$ mM	Dudycha^24^
$K_{i,\text{Ca}}$	Inhibition constant of IP_3_Rs for cytosolic Ca^2+^	1.40 μM	This work
$K_{i,\text{FUM}}$	Inhibition constant of SDH for fumarate	1.3 mM	Cortassa et al.^33^
$K_{i,\text{OAA}}$	Inhibition constant of SDH for oxaloacetate	0.15 mM	Cortassa et al.^33^
$K_{i,\text{NADH}}$	Inhibition constant of IDH for NADH	0.19 mM	Cortassa et al.^33^
$K_{M,\text{AcCoA}}$	Michaelis constant of CS for acetyl-CoA	$1.2614\times 10^{-2}$ mM	Dudycha^24^, Cortassa et al.^33^
$K_{M,\alpha \text{KG}}$	Michaelis constant of KGDH for α-ketoglutarate	1.94 mM	Cortassa et al.^33^
$K_{M,\text{ATPc}}$	Michaelis constant for ATP_c_ hydrolysis due to cellular activity	1 mM	Wacquier et al.^27^
$K_{M,\text{Ca}}$	Michaelis constant of Na^+^/Ca^2+^ exchanger for Ca^2+^	0.375 μM	Cortassa et al.^33^
$K_{M,\text{ISOC}}$	Michaelis constant of IDH for isocitrate	1.52 mM	Dudycha^24^, Cortassa et al.^33^
$K_{M,\text{MAL}}$	Michaelis constant of MDH for malate	0.145 mM	Berndt et al.^25^
$K_{M,\text{Na}}$	Michaelis constant of Na^+^/Ca^2+^ exchanger for Na^+^	9.4 mM	Magnus and Keizer^30^
$K_{M,\text{NAD}}^{\text{IDH}}$	Michaelis constant of IDH for NAD	0.923 mM	Dudycha^24^, Cortassa et al.^33^
$K_{M,\text{NAD}}^{\text{KGDH}}$	Michaelis constant of KGDH for NAD	$3.87\times 10^{-2}$ mM	This work
$K_{M,\text{NAD}}^{\text{MDH}}$	Michaelis constant of MDH for NAD	0.06 mM	Berndt et al.^25^
$K_{M,\text{NADH}}$	Michaelis constant of MDH for NADH	0.044 mM	Cortassa et al.^33^
$K_{M,\text{OAA}}^{\text{CS}}$	Michaelis constant of CS for oxaloacetate	$5\times 10^{-3}$ mM	Berndt et al.^25^, Matsuoka and Srere^64^, Kurz et al.^65^
$K_{M,\text{OAA}}^{\text{MDH}}$	Michaelis constant of MDH for oxaloacetate	0.017 mM	Berndt et al.^25^
$K_{M,\text{SUC}}$	Michaelis constant of SDH for succinate	$3\times 10^{-2}$ mM	Cortassa et al.^33^
$K_{\text{MDH}}$	Equilibrium constant of MDH	$2.756\times 10^{-5}$	Flamholz et al.^29^
$K_{\text{res}}$	Equilibrium constant of O_2_ reduction by NADH in mitochondrial matrix	$1.35\times 10^{18}$	Magnus and Keizer^30^
$K_{s,\text{AcCoA}}$	Other binding constant of citrate synthase for AcCoA	$8.0749\times 10^{-2}$ mM	Dudycha^24^
$K_{\text{SL}}$	Equilibrium constant for SL	0.724	Magnus et al.^29^
$K_{\text{trans}}$	Dissociation constant of mitochondrial uniporter for translocated Ca^2+^	19 μM	Magnus and Keizer^31^
*L*	Equilibrium constant for mitochondrial uniporter conformations	110	Magnus and Keizer^31^
${\left[{\text{Mg}}^{2+}\right]}_{m}$	Mg concentration in the mitochondrial matric	0.4 mM	Cortassa et al.^33^
*n*	Number of Na^+^ binding to electrogenic Na^+^/Ca^2+^ exchanger	3	Magnus and Keizer^30^
$n_{a}$	Mitochondrial uniporter activation cooperativity	2.8	Magnus and Keizer^30^
${\left[{\text{Na}}^{+}\right]}_{c}$	Cytosolic Na^+^ concentration	10 mM	Cortassa et al.^33^
${\left[{\text{Na}}^{+}\right]}_{m}$	Mitochondrial Na^+^ concentration	5 mM	Donoso et al.^66^
$n_{\alpha \text{KG}}$	Hill coefficient of KGDH for αKG	1.2	Cortassa et al.^33^
$n_{i}$	Hill coefficient of IDH for isocitrate	2	Wei et al.^35^
$N_{\text{tot}}$	Total concentration of mitochondrial pyridine nucleotides	0.8 mM	This work
$\left[O_{2}\right]$	O_2_ concentration in mitochondrial matrix	$2.6\times 10^{-5}$ M	Beard^67^
$p_{1}$	Combination of elementary kinetic constants for the 6-state ATPase model	$1.346\times 10^{-8}$	Magnus and Keizer^30^
$p_{2}$	Combination of elementary kinetic constants for the 6-state ATPase model	$7.739\times 10^{-7}$	Magnus and Keizer^30^
$p_{3}$	Combination of elementary kinetic constants for the 6-state ATPase model	$6.65\times 10^{-15}$	Magnus and Keizer^30^
$p_{a}$	Combination of elementary kinetic constants for the 6-state ATPase model	$1.656\times 10^{-5}$ s^−1^	Magnus and Keizer^30^
$p_{c1}$	Combination of elementary kinetic constants for the 6-state ATPase model	$9.651\times 10^{-14}$ s^−1^	Magnus and Keizer^30^
$p_{c2}$	Combination of elementary kinetic constants for the 6-state ATPase model	$4.845\times 10^{-19}$ s^−1^	Magnus and Keizer^30^
${\left[P_{i}\right]}_{c}$	Inorganic phosphate concentration in cytosol	1 mM	Bevington et al.^68^
${\left[P_{i}\right]}_{m}$	Inorganic phosphate concentration in mitochondrial matrix	20 mM	Magnus and Keizer^30^
*R*	Gas constant	8.314 J mol^−1^ K^−1^	
$\rho_{f1}$	Concentration of ATPase pumps	0.23 mM	This work
$\rho_{\text{res}}$	Concentration of H^+^ pumps in mitochondrial membrane	1.00 mM	This work
$r_{1}$	Combination of elementary kinetic constants for the 6-state respiration model	$2.077\times 10^{-18}$	Magnus and Keizer^30^
$r_{2}$	Combination of elementary kinetic constants for the 6-state respiration model	$1.728\times 10^{-9}$	Magnus and Keizer^30^
$r_{3}$	Combination of elementary kinetic constants for the 6-state respiration model	$1.059\times 10^{-26}$	Magnus and Keizer^30^
$r_{a}$	Combination of elementary kinetic constants for the 6-state respiration model	$6.394\times 10^{-10}$ s^−1^	Magnus and Keizer^30^
$r_{c1}$	Combination of elementary kinetic constants for the 6-state respiration model	$2.656\times 10^{-19}$ s^−1^	Magnus and Keizer^30^
$r_{c2}$	Combination of elementary kinetic constants for the 6-state respiration model	$8.632\times 10^{-27}$ s^−1^	Magnus and Keizer^30^
*T*	Temperature	310 K	Cortassa et al.^33^
$V_{\max}^{\text{ANT}}$	Limiting rate of adenine nucleotide translocator (ANT)	4 mM s^−1^	This work
$V_{\max}^{\text{CS}}$	Limiting rate of CS	104 mM s^−1^	This work
$V_{\max}^{\text{IDH}}$	Limiting rate of IDH	1.7767 mM s^−1^	Cortassa et al.^33^
$V_{\max}^{{\text{IP}}_{3}R}$	Limiting release rate of Ca^2+^ through IP_3_Rs	30 s^−1^	This work
$V_{\max}^{\text{KGDH}}$	Limiting rate of KGDH	2.5 mM s^−1^	Cortassa et al.^33^
$V^{\text{LEAK}}$	Leak rate of Ca^2+^ from ER	0.15 s^−1^	This work
$V_{\max}^{\text{MDH}}$	Limiting rate of MDH	128 mM s^−1^	This work
$V_{\max}^{\text{NCX}}$	Limiting rate of Na^+^/Ca^2+^ exchanger	$2\times 10^{-3}$ mM s^−1^	This work
$V_{\max}^{\text{SDH}}$	Limiting rate of SDH	0.5 mM s^−1^	Cortassa et al.^33^
$V_{\max}^{\text{SERCA}}$	Limiting rate of SERCA pumps	0.12 mM s^−1^	Wacquier et al.^27^
$V_{\max}^{\text{UNI}}$	Limiting rate of mitochondrial uniporter	0.30 mM s^−1^	This work

**Table 5:** Experimental values of concentrations, concentration ratios, mitochondrial membrane potential and oscillation period used for the calibration of the model

Variable	Definition	Range (units)	Organism	Reference
ATP/ADP	Total ATP/ADP ratio	[1−3.5]	Astrocytes (mice)	Moein^36^
${\left[{\text{Ca}}^{2+}\right]}_{c}$	Basal and peak Ca^2+^ concentration	0.1 and 1.2μM	Astrocytes (rat)	Pasti et al.^86^, Peuchen et al.^87^
ΔΨ	Mitochondrial membrane potential	[120–170] mV	Astrocytes (human)	Diaz et al.^88^
NADH/NAD	NADH/NAD ratio	0.125 (cellular), 0.2 (mitochondrial)	Astrocytes (mice), liver tissue (rat)	Wilhelm and Hirrlinger^89^, Williamson et al.^90^

To compute their metabolic efficiency, we analyzed mitochondria as out-of-equilibrium chemical engines (Figure 1B) satisfying the second law of thermodynamics:^38^^,^^41^

(Equation 1) $$T\dot{\sigma }=-d_{t}G+{\dot{w}}_{\text{nc}}+{\dot{w}}_{\text{driv}}\;\text{.}$$

Mitochondrial metabolism constitutes an open CRN that continuously harnesses the free energy stored in buffered species (e.g., AcCoA, CoQ, O_2_, $H_{m}^{+}$) to convert cytosolic ATP (ATP_c_) from cytosolic ADP (ADP_c_) while being influenced by ${\text{Na}}^{+}$ homeostasis and cytosolic processes such as Ca^2+^ signaling and ATP_c_ consumption. From a thermodynamic perspective, the synthesis of ATP_c_ and the regulations correspond to free energy exchanges between the mitochondrial engine and its surroundings. They appear in the second law (Equation 1) as the nonconservative work rate, ${\dot{w}}_{\text{nc}}$, and the driving work rate, ${\dot{w}}_{\text{driv}}$, respectively. ${\dot{w}}_{\text{nc}}$ is the energy current maintaining the CRN out of equilibrium while ${\dot{w}}_{\text{driv}}$ is the energy current resulting from the modification of the underlying equilibrium state by the out-of-equilibrium dynamics (Figure 1C). The difference between their sum and the variation in time of the internal Gibbs free energy of mitochondria, G, equals the free energy dissipated by the mitochondrial reactions, i.e., the entropy production rate (EPR) $\dot{\sigma }$ times the absolute temperature *T*.

The expressions of the thermodynamic quantities in Equation 1 are derived for mitochondrial metabolism using a topological analysis^40^^,^^41^^,^^43^ of the corresponding CRN, which allowed us to identify 13 conservation laws and 2 emergent cycles (see subsection Conservation laws and emergent cycles in the STAR Methods). The conservation laws define parts of molecules that remain intact in all mitochondrial reactions and are instrumental to determine the Gibbs free energy G. The emergent cycles are split into 3 effective reactions that can be intuitively identified as mitochondrial output or input

(Equation 2) $${\text{ADP}}_{c}+{\text{Pi}}_{m}\overset{r1_{\text{out}}}{⇌}{\text{ATP}}_{c}+H_{2}O_{m}$$

(Equation 3) $$\frac{3}{22}\;O_{2}+\frac{1}{11}\;\text{AcCoA}+\frac{1}{11}\;\text{CoQ}\overset{r1_{\text{in}}}{⇌}\frac{1}{11}\;\text{CoA}+\frac{2}{11}\;{\text{CO}}_{2}+\frac{1}{11}\;{\text{CoQH}}_{2}$$

(Equation 4) $$H_{m}^{+}+\frac{1}{22}\;O_{2}+\frac{1}{33}\;\text{AcCoA}+\frac{1}{33}\;\text{CoQ}\overset{r2}{⇌}H_{c}^{+}+\frac{1}{33}\;\text{CoA}+\frac{2}{33}\;{\text{CO}}_{2}+\frac{1}{33}\;{\text{CoQH}}_{2}\text{,}$$

with the subscripts c and m referring to the location of some species in the cytosol and mitochondria, respectively. Notice that $r1_{\text{out}}$ and $r1_{\text{in}}$ are not independent as they have the same reaction current and that their sum constitutes the first emergent cycle. In contrast, r2 is the exact chemical equation of the second emergent cycle and has a different reaction current. The nonconservative work rate is correspondingly split into the sum of 3 contributions, equal to the product of the free Gibbs energy of reaction and the effective current of each reaction: ${\dot{w}}_{\text{nc}}={\dot{w}}_{r1_{\text{out}}}+{\dot{w}}_{r1_{\text{in}}}+{\dot{w}}_{r2}$, where ${\dot{w}}_{r1_{\text{out}}}$ quantifies the mitochondrial free energy output corresponding to the synthesis of ATP (Equation 2), while ${\dot{w}}_{r1_{\text{in}}}$ and ${\dot{w}}_{r2}$ quantify the mitochondrial free energy power source in a regime optimizing ATP production - Equation 3 - or the proton driving force - Equation 4 - respectively.

The average thermodynamic efficiency $\bar{\eta }$ of mitochondria can then be calculated as

(Equation 5) $$\bar{\eta }=-\frac{{\bar{w}}_{\text{nc}}^{\text{output}}}{{\bar{w}}_{\text{nc}}^{\text{input}}+{\bar{w}}_{\text{driv}}}$$

where ${\bar{w}}_{\text{nc}}^{\text{input}}={\bar{w}}_{r1_{\text{in}}}+{\bar{w}}_{r2}$ and ${\bar{w}}_{\text{nc}}^{\text{output}}={\bar{w}}_{r1_{\text{out}}}$, and the overline denotes either steady-state quantities or averages over one period of Ca^2+^ oscillations (notice that $\bar{d_{t}G}=0$).

The EPR, nonconservative and driving work contributions vanish at equilibrium according to the second law of thermodynamics but take finite values in nonequilibrium regimes (Figure 1C). The dynamics of the full system (Equations 6, 7, 8, 9, 10, 11, 12, 13, 14, 15, 16, 17, 18, 19, 20, 21, 22, and 23) was simulated for different stimulation conditions and mitochondrial substrate concentrations (i.e., for different $\left[{\text{IP}}_{3}\right]$ and [AcCoA] in the simulations), which allowed for the calculation of the corresponding nonconservative and driving work contributions and, ultimately, of the efficiency of mitochondrial metabolism.

### Ca^2+^-metabolism cross-talk affects the oscillation period and the production of ATP

To calibrate the kinetic model, we compared our simulation results to experimental and simulation data from the literature. More details about the calibration procedure can be found in the STAR Methods. We considered physiological conditions as well as substrate-limited and overstimulation conditions. To provide some baselines, a normal substrate level corresponds to $\left[\text{AcCoA}\right]=10\;\mathrm{\mu M}$^44^ and in the absence of external stimulation of GPCRs (i.e., unstimulated conditions), the so-called basal $\left[{\text{IP}}_{3}\right]$ is close to 0.1μM.^45^ [AcCoA] and $\left[{\text{IP}}_{3}\right]$ are respectively decreased and increased with respect to those baseline values to reach substrate-limited and overstimulation conditions. Substrate-limited conditions lead to energy deficiency (also referred to as ATP_c_ depletion), which we defined as ${\left[\text{ATP}\right]}_{c}$ falling below its physiological value of 1 mM.^46^^,^^47^^,^^48^ Overstimulation of IP_3_Rs by IP_3_ can lead to energy deficiency but is above all characterized by the saturation of the oscillation period or by a high steady-state ${\left[{\text{Ca}}^{2+}\right]}_{c}$ of several hundreds of nanomolars. As described in subsection The relation between mitochondrial efficiency and dissipation is different in fast Ca2+ spiking regimes triggered in substrate-limited or overstimulation conditions, substrate-limited and overstimulation conditions also display different efficiency-dissipation profiles.

**Figure 2:**
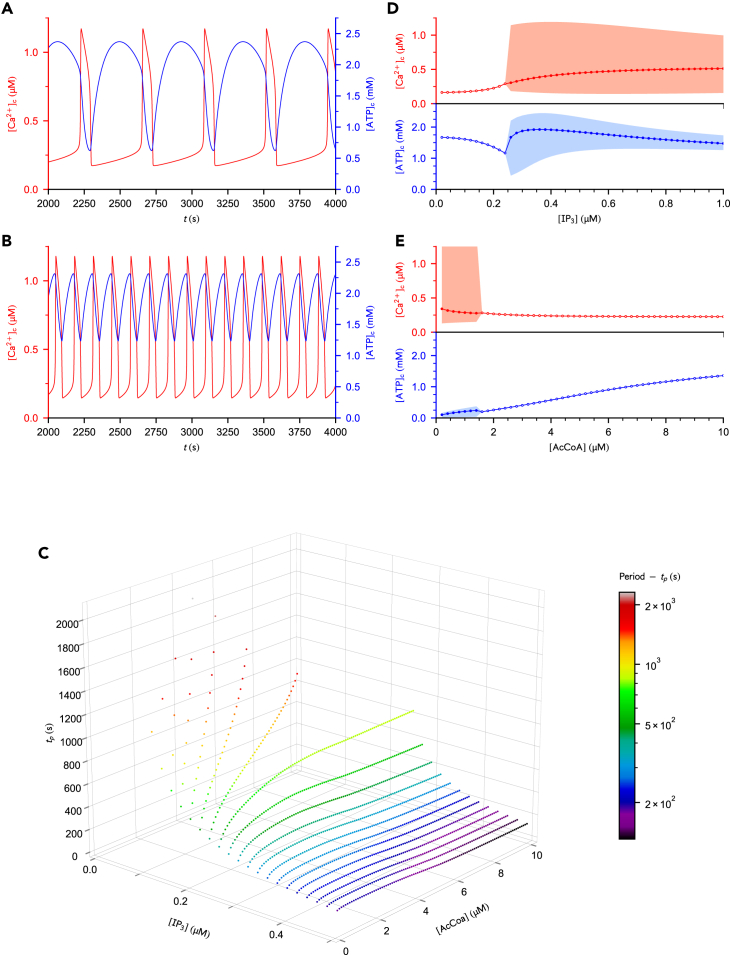
Kinetic behavior of the system (A and B) ${\text{Ca}}_{c}^{2+}$ and ATP_c_ concentrations over time for [AcCoA]=10μM and (*A*) $\left[{\text{IP}}_{3}\right]=0.30\;\mu M$ or (*B*) $\left[{\text{IP}}_{3}\right]=0.50\;\mu M$. (C) Effect of $\left[{\text{IP}}_{3}\right]$ and [AcCoA] on the oscillation period. (D and E) Average concentration of ${\text{Ca}}_{c}^{2+}$ and ATP_c_ as a function of (*D*) $\left[{\text{IP}}_{3}\right]$ for [AcCoA]=10μM or as a function of (*E*) [AcCoA] for $\left[{\text{IP}}_{3}\right]=0.20\;\mu M$. Empty and filled dots represent steady-state and oscillatory regimes, respectively, and the boundaries of the shaded areas correspond to the minimum and maximum concentrations. Parameter values are given in Table 4. Figure S3B illustrates the behavior of ${\left[\text{ATP}\right]}_{c}$ for an extended range of [AcCoA] and $\left[{\text{IP}}_{3}\right]$.

Upon increase of $\left[{\text{IP}}_{3}\right]$ or decrease of [AcCoA], a transition (bifurcation point) from steady-state to slow oscillations marks the onset of the signaling machinery. A decrease in the oscillation period is observed as $\left[{\text{IP}}_{3}\right]$ is increased (Figures 2A–2C) or as [AcCoA] is decreased (Figure 2C). These trends are in agreement with GPCR stimulation experiments performed in various cell types.^49^^,^^50^^,^^51^^,^^52^ They also reproduce the Ca^2+^ dynamics of cultured astrocytes^36^ and *Xenopus* oocytes^53^ reported for limited availability of mitochondrial substrate. In the oscillatory regime, ${\left[\text{ATP}\right]}_{c}$ displays a maximum in dependence on $\left[{\text{IP}}_{3}\right]$ and [AcCoA] (Figures 2D, 2E, and S3), a feature that is also predicted by the model of Wacquier et al*.*^27^ In our simulations, a cusp in the average of ${\left[\text{ATP}\right]}_{c}$ is additionally observed at the critical point (Figures 2D, 2E, and S3). Furthermore, our model predicts, at a more intense stimulation level of GPCRs, a slight decrease in the period as [AcCoA] is increased (Figure 2C, e.g., at $\left[{\text{IP}}_{3}\right]=0.50\;\mathrm{\mu M}$). This result contrasts with the results of Duchen and co-workers who reported that energized mitochondria tend to slow down Ca^2+^ oscillations due to increased mitochondrial uptake and reduction of CICR.^54^^,^^55^ In our model and for those stimulation levels of GPCRs, the increased mitochondrial uptake is accompanied by a larger mitochondrial efflux through NCX, which maintains the activation of CICR (data not shown). Ca^2+^ uptake does not decrease as ΔΨ decreases (Figure S2) indicating that ΔΨ has a minor impact on Ca^2+^ uptake in this model, which can probably not be compared with the chemically induced collapse of ΔΨ reported in the experiments of Duchen et al.^54^^,^^55^

Most of these observations can be rationalized based on the dependence of SERCA pumps on ATP_c_, which enables the switch between ER and mitochondrial Ca^2+^ sequestration and is a key signature of the Ca^2+^-metabolism cross-talk. More precisely, this dependence includes the ATP_c_ consumption associated to Ca^2+^ uptake into the ER and the regulation of SERCA flux ($J_{\text{SERCA}}$) by ATP_c_, which are expressed mathematically in the evolution equation of ${\left[\text{ATP}\right]}_{c}$ (Equation 9) and by the ${\left[\text{ATP}\right]}_{c}$-dependent factor in $J_{\text{SERCA}}$ (Table 2), respectively. From a thermodynamic point of view, $J_{\text{SERCA}}$ contributes to the effective current of $r1_{\text{out}}$ and $r1_{\text{in}}$, hence to mitochondrial thermodynamic efficiency. We stress that in physiological conditions, ${\left[\text{ATP}\right]}_{c}$ is in the millimolar range, which is about 20 times larger than the ATP_c_ dissociation constant of SERCA (Table 4). SERCA’s activity is thus ATP_c_-insensitive but nevertheless impacts ${\left[\text{ATP}\right]}_{c}$ as long as ${\left[{\text{Ca}}^{2+}\right]}_{c}$ is close to or larger than the ${\text{Ca}}_{c}^{2+}$ dissociation constant of SERCA (Table 4). At basal ${\left[{\text{Ca}}^{2+}\right]}_{c}$ (about 0.1μM^54, 55^) SERCA’s activity is thus reduced but is the dominant Ca^2+^ removal mechanism (Figure S1).

**Figure 3:**
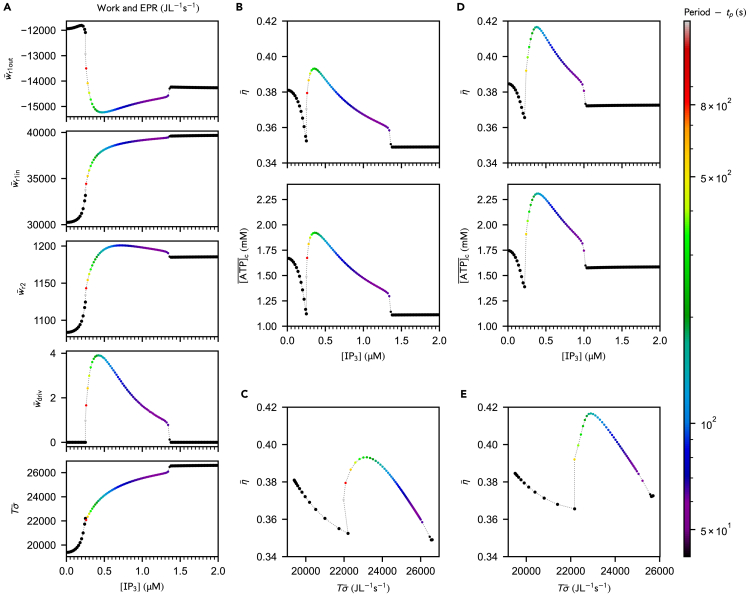
Stimulating the Ca^2+^ signaling machinery impacts the dissipation and efficiency of mitochondrial metabolism via the Ca^2+^-metabolism cross-talk (A) Nonconservative work contributions, driving work and dissipation for different $\left[{\text{IP}}_{3}\right]$. The driving work represents less than 0.017% of the EPR. At high stimulation, oscillations disappear in the favor of a nonequilibrium steady-state regime. (B) Efficiency and ATP_c_ concentration as a function of $\left[{\text{IP}}_{3}\right]$. (C*)* Efficiency as a function of the total dissipation. (D and E) Plots corresponding to (*B*-*C*) for $V_{\max}^{\text{SERCA}}=0.096\;\text{mM}\;s^{-1}$. Empty and filled dots correspond to steady-state or period-averaged quantities, respectively. Unless specified otherwise, parameter values are the same as in Figure 2D. An analogous thermodynamic behavior is observed upon stimulation by AcCoA (Figure S4).

As $\left[{\text{IP}}_{3}\right]$ increases, more Ca^2+^ is released into the cytosol through IP_3_Rs. The steady-state ${\left[\text{ATP}\right]}_{c}$ thus decreases due to a more demanding maintenance of the basal ${\left[{\text{Ca}}^{2+}\right]}_{c}$ via SERCA pumps. At the critical $\left[{\text{IP}}_{3}\right]$ corresponding to the onset of oscillations, mitochondrial sequestration of Ca^2+^ becomes significant, which not only relieves SERCA pumps but also enables the activation of Ca^2+^-sensitive dehydrogenases of the TCA cycle (Figure S1). These combined effects result in an increase of the average ${\left[\text{ATP}\right]}_{c}$. Upon further increase of $\left[{\text{IP}}_{3}\right]$, the activation of mitochondrial dehydrogenases by Ca^2+^ reaches saturation (Figure S1) and the ATP_c_ consumption associated to Ca^2+^ homeostasis is no longer counterbalanced by the Ca^2+^-enhanced mitochondrial activity, which results in a slow decrease in average ${\left[\text{ATP}\right]}_{c}$. Meanwhile, increasing stimulation by IP_3_ favors more frequent opening of the IP_3_Rs, which results in an decrease of the oscillation period. In most mathematical models for Ca^2+^ signaling and in agreement with experimental observations, the oscillation period saturates at high $\left[{\text{IP}}_{3}\right]$^69^ and, beyond a critical $\left[{\text{IP}}_{3}\right]$, oscillations disappear. The cell then exhibits a high-${\left[{\text{Ca}}^{2+}\right]}_{c}$ steady-state,^50^ as reproduced by our simulations (Figure S1). Further stimulation by IP_3_ does not affect the steady-state concentrations reached after termination of the oscillations (see Figure 3B bottom for ${\left[\text{ATP}\right]}_{c}$; Figure S1 for ${\left[{\text{Ca}}^{2+}\right]}_{c}$ and ${\left[{\text{Ca}}^{2+}\right]}_{m}$), suggesting that IP_3_Rs have reached their maximal release rate and contribute to saturation effect.

The impact of AcCoA level on Ca^2+^ oscillations and the presence of the cusp behavior in ${\left[\text{ATP}\right]}_{c}$ is visible at low stimulation by IP_3_, i.e., $\left[{\text{IP}}_{3}\right]\leq 0.24\;\mu M$ (Figures 2C and S3B). In those conditions, oscillations arise for $\left[\text{AcCoA}\right]\leq 5\;\mu M$ (Figure 2C), which correlates with a low ${\left[\text{ATP}\right]}_{c}$ (Figure S3B), and SERCA’s activity is ATP-sensitive (Figure S2). Starting from physiological conditions ($\left[\text{AcCoA}\right]=10\;\mathrm{\mu M}$), ${\left[\text{ATP}\right]}_{c}$ decreases as [AcCoA] is decreased except at the onset of oscillations, where a cusp in ${\left[\text{ATP}\right]}_{c}$ can be observed (Figures 2E and S3D). At this critical point, ${\left[\text{ATP}\right]}_{c}$ becomes limiting for Ca^2+^ uptake by SERCAs and mitochondrial exchanges take over despite a low ΔΨ (Figure S2). Mitochondrial exchanges intensify as [AcCoA] is further decreased (Figure S2) and the larger Ca^2+^ efflux from mitochondria (Figure S2) maintains CICR active, hence the decrease in oscillation period. The amplitude of the cusp is much smaller as compared to the cusp observed with GPCR stimulation because the activation of dehydrogenases by Ca^2+^ plays here a minimal role due to the limited activity of the TCA cycle at low AcCoA level. As shown in Figure S2, the average reaction fluxes of the Ca^2+^-sensitive dehydrogenases ($J_{\text{IDH}}$ and $J_{\text{KGDH}}$) and OXPHOS ($J_{\text{Ox}}$ and $J_{F1}$) decrease monotonically as [AcCoA] decreases. As explained in more detail in subsection Robustness of the efficiency-rescuing effect of Ca2+ oscillations, the *oscillatory* dynamics plays a central role in the rescue of the ATP level because of the phase shift between ER and mitochondrial Ca^2+^ sequestration mechanisms.

To assess the impact of mitochondrial uptake on ΔΨ and mitochondrial metabolism, we inspected the bifurcation diagrams of the mitochondrial uptake flux ($J_{\text{UNI}}$), on the one hand, and of ΔΨ, F1F0-ATPase flux ($J_{F1}$) and respiration flux ($J_{\text{Ox}}$), on the other hand (Figures S1 and S2). For [AcCoA] close to 10 μM and upon increase of $\left[{\text{IP}}_{3}\right]$, the elevation of $J_{\text{UNI}}$ is accompanied by a significant increase in $J_{F1}$, and $J_{\text{Ox}}$, and a slight increase in average ΔΨ, confirming that the activation of the mitochondrial dehydrogenases and the subsequent enhancement of OXPHOS overcomes the depolarization induced by the Ca^2+^ entry (Figure S1). In contrast, a decrease in ΔΨ, $J_{F1}$ and $J_{\text{Ox}}$ is correlated to an increase of $J_{\text{UNI}}$ as [AcCoA] is reduced. However, the decrease in ΔΨ cannot be attributed only to the larger $J_{\text{UNI}}$, but also to the reduced TCA cycle flux ($J_{\text{IDH}}$, $J_{\text{KGDH}}$) feeding the electron transport chain ($J_{\text{Ox}}$) that maintains the polarization of the mitochondrial membrane (Figure S2). In conclusion, the role of Ca^2+^ on metabolism is beneficial upon GPCR stimulation but is potentially inhibitory in substrate-limited conditions.

To summarize, our data suggest that the cusp occurs when ATP_c_ hydrolysis is counterbalanced by the increased ATP production by Ca^2+^-activated mitochondrial metabolism, which occurs when mitochondrial Ca^2+^ uptake becomes significant. The Ca^2+^ build-up activating mitochondrial exchanges and metabolism is either due to CICR (high [AcCoA] and ATP-insensitive SERCA’s activity) or to insufficient Ca^2+^ uptake by SERCA due to ATP_c_ deficiency (low [AcCoA] and ATP-sensitive SERCA’s activity). The role of oscillations in the cusp behavior of ATP_c_ is also to be emphasized. For high [AcCoA] and ATP-insensitive SERCA’s activity, the oscillatory dynamics allows for higher Ca^2+^ levels in the cytosol, which can then be transmitted to mitochondria and sensed by dehydrogenases, without inducing cytotoxic effects. For low [AcCoA] and ATP-sensitive SERCA’s activity, oscillations enable a desynchronization between ER and mitochondrial Ca^2+^ uptake, which intermittently boosts ATP_c_ production while reducing ATP_c_ hydrolysis (see subsection Robustness of the efficiency-rescuing effect of Ca2+ oscillations).

### The efficiency of mitochondrial metabolism displays a maximum in the regime of Ca^2+^ spiking

The nonlinear ATP production observed for different $\left[{\text{IP}}_{3}\right]$ and [AcCoA] (Figures 2D, 2E, and S3*B*) suggests variations in the output work of mitochondria and, possibly, in the thermodynamic efficiency of their metabolism. As confirmed computationally, the output nonconservative work (${\bar{w}}_{r1\text{out}}$) displays a minimum (corresponding to maximal *export* of energy from mitochondria) that coincides with the maximal ${\left[\text{ATP}\right]}_{c}$ in the kinetic simulations (Figures 3A top vs*.* 3B bottom). In the extreme case where oscillations disappear for large stimulation by IP_3_, the efficiency drops and reaches a plateau (Figure 3B top). On the other hand, both the nonconservative input work contributions (${\bar{w}}_{r1\text{in}}$ and ${\bar{w}}_{r2}$) increase with $\left[{\text{IP}}_{3}\right]$ and [AcCoA], while the driving work (${\bar{w}}_{\text{driv}}$) is always negligible compared to the total dissipation (Figures 3A and S4A).

Notably, the maximum in ${\left[\text{ATP}\right]}_{c}$ translates into a maximum in the efficiency of mitochondrial metabolism (Figures 3C and S4B). Such maxima are not systematically observed when [AcCoA] is varied at fixed $\left[{\text{IP}}_{3}\right]$ (Figures S3A and S3B). However, the increase in efficiency at the onset of the oscillatory regime is a robust feature that points to the stabilizing effect of Ca^2+^ spikes on mitochondrial energetics. An increase over a similar range of mitochondrial Ca^2+^ concentration can be induced in an IP_3_-independent way, for example by increasing the leakage of Ca^2+^ from the ER (i.e., increase in $V^{\text{LEAK}}$ with a low $\left[{\text{IP}}_{3}\right]$). In that case, the Ca^2+^ build-up is not accompanied by the emergence of oscillations and does not boost the associated mitochondrial efficiency (Figure S5), which highlights the special role of the *oscillatory* Ca^2+^ dynamics on mitochondrial metabolism in contrast to steady-state regimes.

### The relation between mitochondrial efficiency and dissipation is different in fast Ca^2+^ spiking regimes triggered in substrate-limited or overstimulation conditions

Like in other biological processes such as the migration of molecular motors along microtubules, kinetic proofreading or the regulation of circadian clocks,^70^ the system’s efficiency is maximal at intermediate levels of dissipation, corresponding to a limited range of $\left[{\text{IP}}_{3}\right]$ (Figure 3C).

Overstimulation of the signaling machinery by IP_3_ is counterproductive since it only increases dissipation (Figure 3A bottom). By exploring the behavior of efficiency and EPR at large $\left[{\text{IP}}_{3}\right]$, we observed a saturation effect (Figures 3A–3C) leading to limiting values for the efficiency (≈0.349) and total dissipation ($\approx 26600\;{\text{JL}}^{-1}s^{-1}$). The dependency of efficiency on the total dissipation is highly nonlinear (Figure 3C). Around the onset of oscillations (and for a limited range of dissipation rates), a given dissipation rate can be associated to different efficiencies, in which case the highest efficiency is always reached for the highest Ca^2+^ spiking frequency, while the lowest efficiency corresponds to the steady-state regime. Reversely, different dissipative regimes can yield the same efficiency. In that case, steady-state regimes display the lowest EPR while the fast-spiking regimes are the more dissipative regimes. We hypothesize that in such instances, the selection of the dissipative regime could be guided by constraints imposed by the global energy budget of the cell.

As [AcCoA] is increased, slow-spiking regimes are more dissipative than the fast-spiking ones (Figure S4A, bottom panel) and the efficiency increases almost linearly with dissipation in the oscillatory regime (Figure S4C), which contrasts with the overstimulation by IP_3_. A reason for this difference might be that upon overstimulation by IP_3_, ATP_c_ hydrolysis overbalances the enhanced production of ATP by mitochondrial metabolism, which is not the case at low [AcCoA].

To summarize, fast spiking is less efficient than the slow spiking observed around the bifurcation point, and is also more dissipative when oscillations frequency intensifies due to perturbations in $\left[{\text{IP}}_{3}\right]$.

### Robustness of the efficiency-rescuing effect of Ca^2+^ oscillations

**Figure 4:**
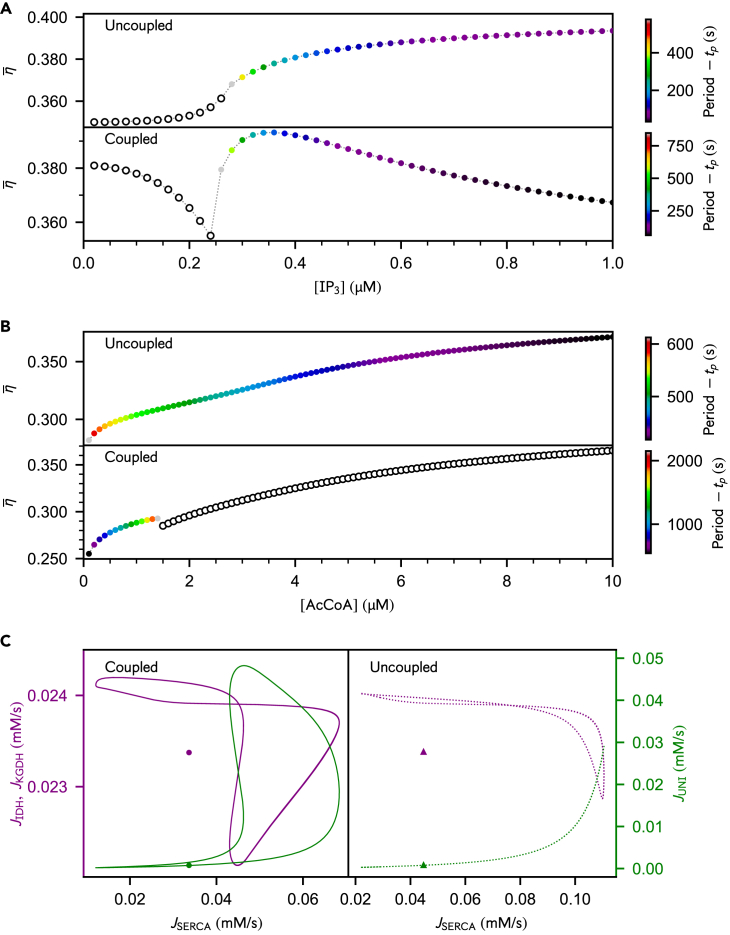
Efficiency of mitochondrial metabolism regulated by Ca^2+^ signaling, Ca^2+^ sequestration fluxes and Ca^2+^-dependent TCA cycle reaction fluxes, without and with the coupling of SERCA pumps to ATP_c_ hydrolysis (A) Stimulating Ca^2+^ release by IP_3_Rs (that is, increasing $\left[{\text{IP}}_{3}\right]$) monotonically increases the efficiency in the uncoupled case, which strongly contrasts with the nonmonotonic dependency on $\left[{\text{IP}}_{3}\right]$ in the coupled case. While varying over different ranges, the period evolves according to the same trends in both cases. (B) Both systems display similar responses in their efficiency upon variations in [AcCoA], but the oscillation period of the uncoupled system is decreasing as [AcCoA] increases. Empty and filled dots correspond respectively to steady-state and oscillatory regimes – note the use of linear colorbar schemes for the period. (C) Phase portraits of Ca^2+^-dependent TCA cycle currents (purple) and mitochondrial Ca^2+^ uptake (green), namely $J_{\text{IDH}}$, $J_{\text{KGDH}}$ and $J_{\text{UNI}}$, vs. ER Ca^2+^ uptake, namely $J_{\text{SERCA}}$. Note that $J_{\text{IDH}}$ and $J_{\text{KGDH}}$ are indistinguishable. Symbols denote steady-state values. Triangles ($\left[{\text{IP}}_{3}\right]=0.24\;\mathrm{\mu M}$) and dotted curves ($\left[{\text{IP}}_{3}\right]=0.32\;\mathrm{\mu M}$) correspond to the uncoupled case, while circles ($\left[{\text{IP}}_{3}\right]=0.18\;\mathrm{\mu M}$) and solid curves ($\left[{\text{IP}}_{3}\right]=0.26\;\mathrm{\mu M}$) represent the coupled case. (*A*) $\left[\text{AcCoA}\right]=10\;\mathrm{\mu M}$, (*B*) $\left[{\text{IP}}_{3}\right]=0.30\;\mathrm{\mu M}$ for the uncoupled case and $\left[{\text{IP}}_{3}\right]=0.20\;\mathrm{\mu M}$ for the coupled case, (*C*) $\left[\text{AcCoA}\right]=1\;\mathrm{\mu M}$. $k_{\text{Hyd}}=1.3\times 10^{-1}$ mM s^−1^ for the uncoupled case. The other parameter values are the same as in Figures 2 and 3.

Our proposed mechanism for the maximum in efficiency relies on the dependence of the SERCA flux on the hydrolysis of ATP_c_ and on the resulting modulation of Ca^2+^ sequestration mechanisms. If Ca^2+^ homeostasis was ATP-independent, Ca^2+^ would always exert a positive feedback on the TCA cycle flux and the efficiency of metabolism would increase monotonically with the Ca^2+^ release from IP_3_Rs. We validated this hypothesis by performing simulations with a modified SERCA flux that is uncoupled from ATP_c_ hydrolysis. The degradation of ATP_c_ then relies exclusively on other ATP-consuming processes mimicking cellular activity (Hyd reaction in Figure 1A). As expected, the Ca^2+^-enhanced ATP production by mitochondria is not restrained upon more intense stimulation by IP_3_ (Figure 4A). This uncoupling also disables the feedback of ATP production by mitochondria on Ca^2+^ oscillations: instead of decreasing, the spike period is barely changed as [AcCoA] decreases (Figure 4B).

In the uncoupled case, $J_{\text{SERCA}}$ is not limited by the depletion of ATP_c_ and both removal mechanisms proceed synchronously, although Ca^2+^ uptake to the ER is predominant (Figure 4C, green dotted curve). While the mitochondrial Ca^2+^ influx $J_{\text{UNI}}$ slightly increases with $J_{\text{SERCA}}$, the Ca^2+^-dependent currents of the TCA cycle, $J_{\text{IDH}}$ and $J_{\text{KGDH}}$ (Figure 4C, purple dotted curves), are barely affected. Upon regulation by ATP_c_ and in substrate-limited conditions, $J_{\text{SERCA}}$ varies over a more restricted range, is on average smaller and proceeds with a slight phase shift with respect to $J_{\text{UNI}}$, which allows for a larger Ca^2+^ influx in mitochondria and a more intense activation of the TCA cycle enzymes (Figure 4C solid curves). This mechanism reinforces the “efficiency-rescuing” role of mitochondrial buffering in substrate-limited conditions.

We also explored the robustness of our results against perturbations in the uptake rate of SERCA pumps of the original model. We mimicked the inhibition of SERCA pumps by decreasing the limiting rate $V_{\max}^{\text{SERCA}}$. The efficiency-dissipation relation displays the same features as in the non-inhibited case (Figures 3C and S4C vs*.* Figures 3E and S4E, respectively). The results of an extended perturbation analysis (Table 6) confirming the robustness of our conclusions are detailed in subsection Sensitivity analysis of the STAR Methods.

**Table 6:** Parametric conditions for each realization of the sensitivity analysis

rea #	$k_{f}^{\text{ACO}}$	$V_{\max}^{\text{ANT}}$	$V_{\text{CS}}^{\max}$	$\rho_{f1}$	$k_{f}^{\text{FH}}$	$g_{H}$	$k_{\text{Hyd}}$	$V_{\max}^{\text{IDH}}$	$V_{\max}^{{\text{IP}}_{3}R}$	$V_{\max}^{\text{KGDH}}$	$V^{\text{LEAK}}$	$V_{\max}^{\text{MDH}}$	$V_{\max}^{\text{NCX}}$	$\rho_{\text{res}}$	$V_{\max}^{\text{SDH}}$	$V_{\max}^{\text{SERCA}}$	$k_{f}^{\text{FH}}$	$V_{\max}^{\text{UNI}}$
**000**	32.2596	0.1642	0.1240	0.2873	0.0022	0.0965	0.2184	1.0192	4.2549	1.0619E-05	108.3939	11.5072	1.7347	2.4632	0.1172	0.5253	8.0114	116.2057
**001**	28.9361	0.1534	0.1096	0.2848	0.0021	0.0927	0.2245	0.9483	3.6600	9.3595E-06	99.4006	11.7411	1.6571	2.6330	0.1256	0.5206	8.5725	135.9840
**002**	30.5150	0.1605	0.1318	0.3071	0.0019	0.0979	0.2482	1.0536	4.0513	1.0947E-05	102.4775	12.6271	1.7147	2.3530	0.1154	0.4575	8.5594	122.5774
**003**	31.0669	0.1554	0.1253	0.2772	0.0022	0.0930	0.2503	0.9669	4.0765	9.9902E-06	110.9615	11.7229	1.6930	2.4687	0.1197	0.4912	8.0044	139.9750
**004**	28.7386	0.1455	0.1189	0.2864	0.0020	0.0825	0.2092	0.9833	4.0047	1.0317E-05	101.1696	11.6808	1.6536	2.5032	0.1282	0.4659	8.7464	116.1729
**005**	28.1559	0.1444	0.1203	0.3194	0.0021	0.0897	0.2305	1.0486	4.0864	9.2162E-06	98.4237	11.2521	1.8507	2.7108	0.1354	0.4726	8.1926	139.9104
**006**	29.6631	0.1564	0.1305	0.2858	0.0021	0.0873	0.2454	0.9942	4.2314	1.0591E-05	110.0077	12.7928	1.7842	2.4806	0.1351	0.4681	8.2690	126.8040
**007**	30.5407	0.1521	0.1207	0.2950	0.0021	0.0990	0.2472	1.0667	4.2996	1.0043E-05	98.4102	13.6152	1.7793	2.4885	0.1318	0.4539	7.5623	122.7604
**008**	28.8359	0.1482	0.1111	0.2974	0.0021	0.0955	0.2267	0.9762	4.3768	1.0999E-05	105.9501	11.6257	1.6276	2.3945	0.1283	0.4530	7.9402	137.5818
**009**	28.9146	0.1610	0.1169	0.2757	0.0019	0.0831	0.2509	1.0335	3.7614	1.0627E-05	103.0207	13.2424	1.8459	2.3296	0.1385	0.4759	8.6330	126.7602
**ref**	30.0000	0.1500	0.1200	0.3000	0.0020	0.0900	0.2300	1.0000	4.0000	1.000E-05	104.0000	12.5000	1.7767	2.5000	0.1250	0.5000	8.3000	128.0000

All leading constants are altered by a random perturbation comprised between −10% and 10% of the parameter reference value (**ref**) of Table 4.

Together, these results confirm that the cross-talk between Ca^2+^ signaling and mitochondrial energy metabolism is a major mechanism underlying the maximum in efficiency arising in the spiking regime, even when the amplitude of this coupling is reduced due to the inhibition of SERCA pumps.

## Discussion

Here, we examined the impact of Ca^2+^ signaling on the efficiency of mitochondrial metabolism by using tools from the nonequilibrium thermodynamics of CRNs on a detailed and experimentally calibrated kinetic model of the Ca^2+^-metabolism cross-talk. Our results highlight that, despite a usually higher dissipation rate compared to steady-state regimes, Ca^2+^ oscillations can enhance the efficiency of mitochondrial metabolism. Increasing stimulation by IP_3_ or inducing energy deficiency by decreasing the mitochondrial AcCoA level reduces the steady-state efficiency of metabolism, but at the onset of oscillations, the efficiency raises with a cusp-like transition and reaches a maximum of about 40% before decreasing again at higher stimulation/lower AcCoA level. This value is in the range of the efficiencies of the TCA cycle and OXPHOS (30% and 42%, respectively) estimated in the absence of regulation with a nonequilibrium thermodynamic approach.^16^ Moreover, slow-spiking is less dissipative than fast-spiking. Thus, we hypothesize that, for a given cell state, there exists an optimal stimulation level leading to slow-spiking/low-dissipation oscillations which maximize the efficiency of metabolism during signaling. For higher stimulation, the Ca^2+^ signaling machinery then generates more dissipative regimes of gradually decreasing efficiency.

In the broader context of physical bioenergetics, energetic costs are usually assessed by evaluating the Gibbs free energy of reaction ($\Delta_{r}G$) dissipated or the equivalent number of ATP molecules produced/consumed along the processes of interest.^8^^,^^14^^,^^15^ However, such purely thermodynamic approaches do not account for reaction kinetics and thus cannot quantify the rates of free energy transduction and dissipation. Significant efforts have been made in the direction of adding thermodynamic constraints in flux balance analysis of metabolic networks.^71^^,^^72^ A few attempts have also been made to account for more complex kinetic effects such as enzyme saturation, leading to insights into the trade-offs between energy production and enzyme costs in glycolysis.^12^^,^^73^^,^^74^ Nevertheless, all these approaches rely on optimized nonequilibrium steady-states, which may not correspond to physiological conditions and cannot capture the energetic impact of time-dependent behaviors, such as the energy-rescuing effect of Ca^2+^ oscillations quantified here. Our approach overcomes these limitations, based on the rigorous thermodynamic analysis of a curated dynamical model. Due to the modular structure of the model, our approach can be extended with additional pathways, such as glycolysis or one-carbon metabolism, with the aim to perform *integrative modeling* of cell metabolism.

Finally, a key element of the metabolic efficiency management in the presence of Ca^2+^ regulation is the possibility for Ca^2+^ oscillations, as an increase in the steady-state Ca^2+^ level is not sufficient to boost mitochondrial efficiency. Ca^2+^ oscillations are shaped by the interplay between SERCA and mitochondrial uptakes. Alterations in Ca^2+^ removal mechanisms due to mutations, generation of reactive oxygen species or remodeling of channel and pump expression are ubiquitous in pathological states such as mitochondrial^75^ and neurodegenerative diseases,^76^^,^^77^ cancer^78^ or diabetes,^79^ and therapeutic strategies targeting Ca^2+^ homeostasis and signaling have started to emerge.^80^^,^^81^ Some of these changes can be captured by perturbations in the kinetic parameters of the Ca^2+^ fluxes,^82^ which would make the use of our approach in the context of disease quite straightforward. Overall, our methodology thus paves the way for a more systematic characterization of the dynamical energetic impact of metabolism regulation, which could improve the current understanding of pathway selection mechanisms in health and disease.

### Limitations of the study

A limitation of the study is related to the scarcity of kinetic data available per cell and organism. The computational model was carefully calibrated on astrocyte data but a wide range of kinetic parameters (Table 4) was collected from previous models based on different cell types and mammalian organisms. Ideally, the kinetic consistency of the model could be improved by using parameters characterized in the same cell line and experimental conditions, bearing in mind that *in vitro* or *in vivo* data may also differ.^83^ Another limitation is that the rate of ATP consumption by cellular activity other than SERCA’s is a highly coarse-grained estimation, although widely used in Ca^2+^ modeling. Ongoing research about the energetic costs in biology might provide more accurate values in the future. Finally, the experimental verification of our modeling predictions on the thermodynamic efficiency is challenging, since it requires the dynamic measurement of fluxes and Gibbs free energies of reactions, independently of competing metabolic processes.

## STAR★Methods

### Key resources table

**Table:** 

REAGENT or RESOURCE	SOURCE	IDENTIFIER
**Deposited data**		

eQuilibrator	https://equilibrator.weizmann.ac.il/	https://doi.org/10.1093/nar/gkr874

**Software and algorithms**		

Simulation algorithms	Zenodo or https://gitlab.lcsb.uni.lu/ICS-lcsb/net-ca-mito.git	https://doi.org/10.5281/zenodo.10530533
Python version 3.9.13	Python Software Foundation	https://www.python.org
eQuilibrator	https://equilibrator.weizmann.ac.il/	https://doi.org/10.1093/nar/gkr874

### Resource availability

#### Lead contact

Further information and requests for resources should be directed to and will be fulfilled by the lead contact, Alexander Skupin (alexander.skupin@uni.lu).

#### Materials availability

This study did not generate new unique reagents.

#### Data and code availability

Transformed Gibbs free energies of reaction were retrieved from eQuilibrator.^29^ All original code^85^ has been deposited at Zenodo and https://gitlab.lcsb.uni.lu/ICS-lcsb/net-ca-mito.git and is publicly available as of the date of publication. The DOI is listed in the key resources table. Data were generated by running the simulation algorithms in Python (version 3.9.13). Any additional information required to reanalyze the simulation data of this paper is available from the lead contact upon request.

### Method details

#### Kinetic model

##### Mitochondrial reactions

Our model for mitochondrial metabolism is partly based on the pioneering work of Magnus and Keizer.^24−26.^ In particular, the synthesis of ATP by F1F0-ATPase and the oxidation of NADH into NAD by the electron transport chain are both accompanied by a flux of protons across the mitochondrial membrane. While the reaction fluxes and the proton fluxes are described by slightly different expressions in the Magnus-Keizer model, we consider that the proton flux is a multiple of the reaction flux, and fix the proportionality coefficient according to the known stoichiometry (see Table 1), as done in previous models.^27^,^34^ The Magnus-Keizer model accounts for mitochondrial carbon metabolism in a compact way, with effective fluxes for the TCA cycle and the associated generation of high energy electrons in the form of NADH. These fluxes have a constant component representing a basal flux, which is non-zero even in the absence of carbon input (glucose), and a contribution from glycolysis, which is assumed to be essentially captured by the flux through PDH.^31^ Although these effective fluxes depend on ${\left[{\text{Ca}}^{2+}\right]}_{m}$ to account for the ${\text{Ca}}^{2+}$-regulation of PDH, they do not reflect the activatory role of ${\text{Ca}}^{2+}$ on IDH and KGDH. We thus replace these effective contributions by incorporating the more detailed model of the TCA cycle from Dudycha et al.^24^ A reaction current is associated to each of the 8 enzymatic reaction of the TCA cycles, which are subjected to possible regulations by ${\text{Ca}}^{2+}$, but also by ${\text{Mg}}^{2+}$, ATP, NADH, and others. Additionally, Dudycha’s model accounts for the aspartate aminotransferase reaction, which is not part of the TCA cycle *per se*, but converts oxaloacetate and glutamate into α-ketoglutarate and aspartate. We neglect the aspartate aminotransferase reaction to focus exclusively on the TCA reactions. Finally, we use acetyl-CoA (AcCoA) at the entry of the TCA cycle as the carbon input of the mitochondrial metabolism instead of glucose at the entry of glycolysis like in Magnus-Keizer model. Note that these modifications are similar to the ones adopted by Cortassa et al.^33^

##### Cytosolic reactions and calcium signaling

Cortassa et al. treated ${\left[{\text{Ca}}^{2+}\right]}_{c}$ as a constant parameter and neglected the ATP-consuming ${\text{Ca}}^{2+}$ exchanges between the endoplasmic reticulum (ER) and the cytosol.^33^ Their resulting model can therefore not fully capture the cross-talk between mitochondrial ATP production and ${\text{Ca}}^{2+}$ signaling. To close this gap, our model explicitly describes the coupling between ATP hydrolysis and the uptake of ${\text{Ca}}^{2+}$ into the ER by SERCA pumps as done by Wacquier et al.^27^ More precisely, the SERCA flux is ATP-dependent and affects the cytosolic concentration of ATP. The ${\text{Ca}}^{2+}$ uptake by SERCAs is balanced by the efflux of ${\text{Ca}}^{2+}$ from the ER through ${\text{IP}}_{3}$ receptors (${\text{IP}}_{3}$Rs) and passive leak through the membrane of the ER. The flux through ${\text{IP}}_{3}$Rs is modeled by a function depending solely on the concentrations of ${\text{IP}}_{3}$ and cytosolic ${\text{Ca}}^{2+}$, as in previous models.^26^

##### Fluxes and evolution equations

Overall, we consider 32 species and 17 chemical reactions taking place in or between compartments corresponding to the mitochondrial matrix, the cytosol and the ER. The chemical equations describing these reactions can be found in Table 1. A current $J_{\kappa }$ (given in Table 2) is associated to each chemical reaction κ and is normalized with respect to the volume of the corresponding cell compartment (cytosol, ER or mitochondria). The concentration of the controlled species {Pi_c_, Pi_m_, ${\text{Na}}_{c}^{+}$, ${\text{Na}}_{m}^{+}$, IP_3_, $H_{c}^{+}$, $H_{m}^{+}$, O_2_, H_2_O_c_, H_2_O_m_, AcCoA, CoA, CO_2_, CoQH_2_, CoQ} is fixed in time (see also Figure 1 in the main text), meaning that the effect of chemical reactions is balanced by additional processes which are not described explicitly in the model. The rate equations for the other species are given in Equations 6, 7, 8, 9, 10, 11, 12, 13, 14, 15, 16, 17, 18, 19, 20, 21, 22, and 23. Stoichiometric and volumetric coefficients are included in Equations 6, 7, 8, 9, 10, 11, 12, 13, 14, 15, 16, 17, 18, 19, 20, 21, 22, and 23 to guarantee mass balance across cell compartments. Specifically, $\alpha =V_{\text{ER}}/V_{c}$ and $\delta =V_{m}/V_{c}$, where $V_{c}$, $V_{\text{ER}}$ and $V_{m}$ are the volumes of the cytosol, of the ER and of mitochondria, respectively. Ca^2+^ buffering in these compartments is accounted for by the coefficients $f_{c}$, $f_{\text{ER}}$ and $f_{m}$, which correspond to the fraction of free Ca^2+^ in the compartment of interest. Finally, we mention that all fluxes are expressed in mM/s and concentration units are mM, except for Ca^2+^ concentrations which are in μM, and coefficient $\gamma =10^{3}$ μM/mM is therefore introduced to ensure consistency in units.

(Equation 6) $$d_{t}{\left[\text{ADP}\right]}_{c}=-\delta J_{\text{ANT}}+J_{\text{Hyd}}+\frac{1}{2}J_{\text{SERCA}}$$

(Equation 7) $$d_{t}{\left[\text{ADP}\right]}_{m}=J_{\text{ANT}}-J_{F1}-J_{\text{SL}}$$

(Equation 8) $$d_{t}{\left[\alpha \text{KG}\right]}_{m}=J_{\text{IDH}}-J_{\text{KGDH}}$$

(Equation 9) $$d_{t}{\left[\text{ATP}\right]}_{c}=\delta J_{\text{ANT}}-J_{\text{Hyd}}-\frac{1}{2}J_{\text{SERCA}}$$

(Equation 10) $$d_{t}{\left[\text{ATP}\right]}_{m}=-J_{\text{ANT}}+J_{F1}+J_{\text{SL}}$$

(Equation 11) $$d_{t}{\left[{\text{Ca}}^{2+}\right]}_{c}=\frac{f_{c}}{\gamma }\left(-J_{\text{SERCA}}+J_{\text{ERout}}+\delta \left(J_{\text{NCX}}-J_{\text{UNI}}\right)\right)$$

(Equation 12) $$d_{t}{\left[{\text{Ca}}^{2+}\right]}_{m}=\frac{f_{m}}{\gamma }\left(J_{\text{UNI}}-J_{\text{NCX}}\right)$$

(Equation 13) $$d_{t}{\left[{\text{Ca}}^{2+}\right]}_{\text{ER}}=-\frac{f_{\text{ER}}}{\gamma \alpha }\left(J_{\text{SERCA}}-J_{\text{ERout}}\right)$$

(Equation 14) $$d_{t}{\left[\text{CIT}\right]}_{m}=J_{\text{CS}}-J_{\text{ACO}}$$

(Equation 15) $$d_{t}{\left[\text{FUM}\right]}_{m}=J_{\text{SDH}}-J_{\text{FH}}$$

(Equation 16) $$d_{t}{\left[\text{ISOC}\right]}_{m}=J_{\text{ACO}}-J_{\text{IDH}}$$

(Equation 17) $$d_{t}{\left[\text{MAL}\right]}_{m}=J_{\text{FH}}-J_{\text{MDH}}$$

(Equation 18) $$d_{t}{\left[\text{NAD}\right]}_{m}=J_{\text{Ox}}-J_{\text{IDH}}-J_{\text{KGDH}}-J_{\text{MDH}}$$

(Equation 19) $$d_{t}{\left[\text{NADH}\right]}_{m}=-J_{\text{Ox}}+J_{\text{IDH}}+J_{\text{KGDH}}+J_{\text{MDH}}$$

(Equation 20) $$d_{t}{\left[\text{OAA}\right]}_{m}=J_{\text{MDH}}-J_{\text{CS}}$$

(Equation 21) $$d_{t}\Delta \Psi =\frac{1}{C_{m}}\left(10\;J_{\text{Ox}}-3\;J_{F1}-J_{\text{ANT}}-J_{\text{Hl}}-J_{\text{NCX}}-2\;J_{\text{UNI}}\right)$$

(Equation 22) $$d_{t}{\left[\text{SCoA}\right]}_{m}=J_{\text{KGDH}}-J_{\text{SL}}$$

(Equation 23) $$d_{t}{\left[\text{SUC}\right]}_{m}=J_{\text{SL}}-J_{\text{SDH}}\text{.}$$

##### Model calibration

Except for the leading constants ($V_{\max}$, ρ and $k_{f}$), most parameter values of our model were taken from previous models. The calibration of our model thus consisted in finding a set of $V_{\max}$, ρ and $k_{f}$ generating data in agreement with experimental data according to the following criteria:

1. negative Gibbs free energy of reaction for all processes (thermodynamic consistency);
2. realistic concentrations, concentration ratios and mitochondrial membrane potential for physiological conditions ([AcCoA] = 10μM and $\left[{\text{IP}}_{3}\right]$ = 0.1μM) based on experimental data from different mammalian cell types (Table 5);
3. reproducing key trends (in ATP:ADP and period of Ca^2+^ oscillations) in response to GPCR stimulation and mitochondrial substrate availability, characteristic of the Ca^2+^-metabolism cross-talk, based on data from cultured astrocytes.

Thermodynamic consistency was satisfied for all bifurcation diagrams, with a notable exception for Gibbs free energy associated to SERCA in the uncoupled case. Indeed, since SERCA’s activity is modeled as independent from ATP_c_ hydrolysis in the uncoupled case and Ca^2+^ physiologically more abundant in the ER than in the cytosol, a positive Gibbs free energy of reaction is to be expected. We emphasize that the uncoupled case is unphysiological.

The bidirectional feedback between Ca^2+^ signaling and mitochondrial metabolism has recently been investigated in C8-D1A astrocytes^36^ and our model was calibrated to reproduce the following trends:

1. decreasing the glucose concentration in the cell culture medium decreases the ATP:ADP ratio;
2. increasing the stimulation of GPCRs decreases the period of Ca^2+^ oscillations;
3. decreasing the glucose concentration in the medium decreases the period of Ca^2+^ oscillations;
4. increasing the stimulation of GPCRs increases the ATP:ADP ratio.

The impact of $\left[{\text{IP}}_{3}\right]$ and [AcCoA] on the period of Ca^2+^ oscillations (Figure 2C of the manuscript) is to be compared with the experimental data of Figures 4.13 (A) and 4.10 (B-C) of Moein,^36^ respectively. The evolution of the ATP:ADP ratio in dependence of $\left[{\text{IP}}_{3}\right]$ and [AcCoA] (Figures S1 - before overstimulation - and S2) can be compared to Figures 4.17 and 4.12 (B) of Moein,^36^ respectively.

#### Concepts of biothermodynamics

##### Definitions

The entropy production rate (EPR) associated to a chemical reaction ρ is given by

(Equation 24) $${\dot{\sigma }}_{\rho }=-\frac{J_{\rho }\;\Delta_{r}G_{\rho }}{T}\geq 0\text{,}$$

where *T* is the absolute temperature while $J_{\rho }$ and $\Delta_{r}G_{\rho }$ are the current and Gibbs free energy of reaction ρ, respectively. In nonequilibrium thermodynamics, $-\Delta_{r}G_{\rho }$ is the *force* driving the reaction while $J_{\rho }$ is the reaction *flux* resulting from this force. The equilibrium state is characterized by zero forces and hence zero fluxes.

The Gibbs free energy of reaction ρ is defined by^91^

(Equation 25) $$\Delta_{r}G_{\rho }=\sum_{i}S_{i}^{\rho }\mu_{i}\text{,}$$

where $S_{i}^{\rho }$ is the net stoichiometric coefficient of species *i* in reaction ρ and $\mu_{i}$ is the chemical potential of species *i*. Under the hypothesis of *local equilibrium*, *i.e*., state variables such as temperature and pressure relax to equilibrium on a much faster timescale than chemical reactions, the expressions for the chemical potentials derived at equilibrium still hold locally out-of-equilibrium.^91^ The chemical potential $\mu_{i}$ is thus given by

(Equation 26) $$\mu_{i}=\mu_{i}^{\circ }+RT\;\ln\;a_{i}\text{,}$$

where $\mu_{i}^{\circ }$ and $a_{i}$ denote the standard chemical potential and the activity of species *i*, respectively. The activity accounts for the interactions between chemical species present in solution and is related to the concentration by the coefficient of activity $\gamma_{i}$, which depends on the ionic strength,^92^ *I*, such that $a_{i}=\gamma_{i}\frac{\left[i\right]}{c^{\circ }}$, where [i] and $c^{\circ }$ are the concentration of species *i* and standard concentration, respectively. In ideal solutions, $\gamma_{i}=1$. Standard conditions correspond to atmospheric pressure $p^{\circ }=1$ bar and molar concentrations $c^{\circ }=1\;M$.

Standard chemical potentials are directly related to the standard Gibbs free energy of reaction $\Delta_{r}G_{\rho }^{\circ }=\sum_{i}S_{i}^{\rho }\mu_{i}^{\circ }=-RT\;\ln\;K_{\rho }$, where $K_{\rho }=\prod_{i}a_{i,eq}^{S_{i}^{\rho }}$ is the equilibrium constant of reaction ρ. Hence, $\Delta_{r}G_{\rho }$ can then be rewritten as

(Equation 27) $$\Delta_{r}G_{\rho }=\Delta_{r}G_{\rho }^{\circ }+RT\;\ln\prod_{i}a_{i}^{S_{i}^{\rho }}=RT\;\ln\prod_{i}{\left(\frac{a_{i}}{a_{i,eq}}\right)}^{S_{i}^{\rho }}\text{.}$$

From a practical point of view, standard Gibbs free energies of reaction and activity coefficients are usually available in thermodynamic tables. As described in the next subsection, further adaptations can be done to describe more adequately the physiological environment in which biochemical reactions take place.

##### Physiological conditions

Cells are compartmentalized into specialized organelles whose composition can widely differ. For example, mitochondrial and cytosolic pH are 8^60^ and 7.2,^59^ respectively. A plethora of buffering mechanisms regulate their internal concentration of metallic ions and pH, and thereby ensures the maintenance of homeostasis. Some chemical species can exist in different forms, that is, bound to metallic cations or at different levels of protonation (for example, “ATP” can be ${\text{ATP}}^{4-}$, ${\text{HATP}}^{3-}$, ${\text{MgATP}}^{2-}$, *etc.*), and their relative abundance depends on the internal environment of the organelle. For the sake of compactness, biochemical reactions are thus usually written in terms of *pseudoisomers*, that is, without explicitly mentioning the state of the species and without detailing the consumption or production of protons/metallic ions by the reaction (e.g. $\text{ATP}+H_{2}O⇌\text{ADP}+P_{i}$).^92^

To describe biochemical reactions from a thermodynamic point of view, their associated standard Gibbs free energy can be rescaled to match the equilibrium corresponding the physiological pH and metallic ions concentrations, but also incorporates the activity coefficients corresponding to a physiological ionic strength (I=0.120M^28^^,^^92^). The resulting $\Delta_{r}G_{\kappa }^{\prime \circ }$ is subsequently used to calculate the transformed Gibbs free energy of reaction

(Equation 28) $$\Delta_{r}G_{\kappa }^{\prime }=\Delta_{r}G_{\kappa }^{\prime \circ }+RT\;\ln\prod_{j}{\left[j\right]}^{S_{j}^{\kappa }}\text{,}$$

where [j] is the concentration of pseudoisomer *j* and $S_{j}^{\kappa }$ is the net stoichiometric coefficient of pseudoisomer *j* in reaction κ. Complementary approaches^93^^,^^94^ have led to the development of databases^29^^,^^95^ from which we retrieved $\Delta_{r}G_{\kappa }^{\prime \circ }$ for different pH, ionic strength and metallic ion concentrations.

Electrogenic processes, such as ${\text{Ca}}^{2+}$ exchanges, the exchange of ${\text{ATP}}^{4-}$ and ${\text{ADP}}^{3-}$ *via* the antiporter and the transfer of protons accompanying oxidative phosphorylation, constitute notable exceptions where the charges of species need to be explicitly accounted for. Indeed, electrostatic interactions affect the Gibbs free energy and in that case, the right-hand side of Equation 28 must also comprise the term related to the electric potential in the compartment of interest. We thus distinguish the charged species {i} from the pseudoisomers {j}. More precisely, $\Delta_{r}G_{\kappa }^{\prime }=\sum_{j}S_{j}^{\kappa }\mu_{j}+\sum_{i}S_{i}^{\kappa }{\bar{\mu }}_{i}$ where ${\bar{\mu }}_{i}$ is the *electrochemical potential*

(Equation 29) $${\bar{\mu }}_{i}=\mu_{i}^{\circ }+RT\;\ln\;a_{i}+z_{i}FV_{r\left(i\right)}\text{,}$$

where $z_{i}$ is the charge of species *i* (for example, z=+2 for ${\text{Ca}}^{2+}$), *F* is the Faraday constant and $V_{r\left(i\right)}$ is the electric potential in the compartment *r* where species *i* is considered. Overall, this leads to

(Equation 30) $$\Delta_{r}G_{\kappa }^{\prime }=\Delta_{r}G_{\kappa }^{\prime \circ }+RT\;\ln\prod_{j}{\left[j\right]}^{S_{j}^{\kappa }}+RT\;\ln\prod_{i}{\left[i\right]}^{S_{i}^{\kappa }}+\sum_{i}S_{i}^{\kappa }z_{i}FV_{r\left(i\right)}\text{.}$$

When a charged species is exchanged between two compartments, the $\Delta_{r}G_{\kappa }^{\prime }$ associated to this transport process depends on the difference of potential between the two compartments. For example, if we consider the transport of ${\text{Ca}}^{2+}$ from cytosol to mitochondria, $\Delta_{r}G_{\kappa }^{\prime }=RT\;\ln\frac{{\left[{\text{Ca}}^{2+}\right]}_{m}}{{\left[{\text{Ca}}^{2+}\right]}_{c}}+2F\left(V_{m}-V_{c}\right)=RT\;\ln\frac{{\left[{\text{Ca}}^{2+}\right]}_{m}}{{\left[{\text{Ca}}^{2+}\right]}_{c}}-2F\Delta \psi$.

Although pH (and hence proton concentrations) in mitochondria and cytosol are assumed to be constant due to strong buffering, proton transfer across mitochondrial membrane still affects the membrane potential (at least, at the local scale that is considered in the present model), which in turns affect $\Delta_{r}G_{\kappa }^{\prime }$. The expressions for the $\Delta_{r}G_{\kappa }^{\prime }$ of each process of the model can be found in Table 3.

#### Nonequilibrium thermodynamic analysis

##### Mitochondria as chemical engines

Mitochondria can be considered as engines converting ADP_c_ into ATP_c_ *via* 11 so-called *internal reactions*

$$\kappa_{i}\in \left\{\text{ANT},F1,\text{OX},\text{CS},\text{ACO},\text{IDH},\text{KGDH},\text{SL},\text{SDH},\text{FH},\text{MDH}\right\}\text{,}$$

modeling mitochondrial metabolism, which are coupled to 6 *external reactions*

$$\kappa_{e}\in \left\{\text{ERout},\text{SERCA},\text{NCX},\text{UNI},\text{HYD},\text{Hl}\right\}\text{,}$$

representing Ca^2+^ signaling, cell activity and ionic homeostasis. The chemical species involved in internal reactions are categorized into two groups, referred to as *internal species*

$$X\in \left\{{\text{ATP}}_{m},{\text{ADP}}_{m},\text{NADH},\text{NAD},\text{OAA},\text{CIT},\text{ISOC},\alpha \text{KG},\text{SCoA},\text{SUC},\text{FUM},\text{MAL}\right\}\text{,}$$

and *exchanged species*

$$Y\in \left\{{\text{ATP}}_{c},{\text{ADP}}_{c},{\text{Pi}}_{m},H_{c}^{+},H_{m}^{+},O_{2},H_{2}O_{m},\text{AcCoA},\text{CoA},{\text{CO}}_{2},\text{CoQ},{\text{CoQH}}_{2}\right\}\text{.}$$

The former is the set of species involved only in the internal reactions, while the latter includes the controlled species and the species involved also in the external reactions. Thus, the rate equations for internal and exchanged species can be respectively written as

(Equation 31a) $$d_{t}\left[X\right]=\sum_{\kappa_{i}}S_{\kappa_{i}}^{X}J_{\kappa_{i}}\text{,}$$

(Equation 31b) $$\frac{V_{\text{ref}}^{Y}}{V_{m}}d_{t}\left[Y\right]=\sum_{\kappa_{i}}S_{\kappa_{i}}^{Y}J_{\kappa_{i}}+I^{Y}\text{,}$$

where $V_{\text{ref}}^{Y}$ is the volume of the compartment to which species Y belongs, and $I^{Y}$ is the exchange current either accounting for the external reactions (named flux control^42^) or modeling additional processes that are responsible for the homeostasis of the controlled species (named concentration control^42^). On the one hand ${\text{ATP}}_{c}$ and ${\text{ADP}}_{c}$ are involved in the external reactions SERCA and HYD and hence $I^{{\text{ATP}}_{c}}=-I^{{\text{ADP}}_{c}}=\frac{1}{\delta }\left(-\frac{1}{2}J_{\text{SERCA}}-J_{\text{HYD}}\right)$; on the other hand, the other exchanged species (*i.e.*, ${\text{Pi}}_{m}$, $H_{m}^{+}$, $H_{m}^{+}$, $O_{2}$, $H_{2}O_{m}$, AcCoA, CoA, ${\text{CO}}_{2}$, ${\text{CoQH}}_{2}$, CoQ) are controlled species and hence $I^{Y}=-\sum_{\kappa_{i}}S_{\kappa_{i}}^{Y}J_{\kappa_{i}}$.

##### Second law for mitochondrial metabolism

In general, the second law of thermodynamics for open CRNs can be written as^38^^,^^41^

(Equation 32) $$T\dot{\sigma }=-d_{t}G+{\dot{w}}_{\text{nc}}+{\dot{w}}_{\text{driv}}\text{,}$$

where G is the (semi-grand) *Gibbs free energy* of the system, while ${\dot{w}}_{\text{nc}}$ and ${\dot{w}}_{\text{driv}}$, respectively referred to as the *nonconservative work rate* and the *driving work rate*, are related to the energetic cost of maintaining CRNs out of equilibrium *via* the exchange of species {Y}. Since G is a state function, its time derivative vanishes at steady state as well as when averaged over one period in the case of an oscillatory regime.

In the following, we use a topological analysis^38^^,^^41^ to derive the explicit expressions of the nonconservative work rate and the driving work rate for mitochondrial metabolism.

###### Remark

The rate Equations 6, 7, 8, 9, 10, 11, 12, 13, 14, 15, 16, 17, 18, 19, 20, 21, 22, and 23 are coarse-grained, namely, each reactive process represents a sequence of out-of-equilibrium elementary reactions involving intermediate species whose dynamical behavior is not described. Each of these elementary reactions might affect the energetics of the whole system. Nevertheless, under the assumption of the existence of a time scale separation between the evolution of the species accounted by the dynamical model and the coarse-grained intermediate species, our thermodynamic analysis characterizes the correct energetics of the whole system, as proven elsewhere.^40^^,^^43^

##### Conservation laws and emergent cycles

For our model, the *stoichiometric matrix* S encoding the net stoichiometric coefficients of internal species X and exchanged species Y in the internal reactions $\kappa_{i}$ reads(Equation 33)

The 13 (linearly-independent) left-null eigenvectors of S, encoded as rows of the matrix(Equation 34)which therefore satisfies LS=0, define the conservation laws. Indeed, for the every row λ of L (labeled using chemical symbols in Equation 34 for reasons that will be explained in the following section), the quantity $L^{\lambda }=\sum_{X}L_{X}^{\lambda }\left[X\right]+\sum_{Y}L_{Y}^{\lambda }\left[Y\right]$ would be a conserved quantity if mitochondria were closed systems, namely, if $I^{Y}=0$ ∀Y. When $I^{Y}\neq 0$, only 3 out of the 13 conservation laws corresponding to the last three rows of L in Equation 34 involve exclusively internal species and their corresponding quantities $L^{\lambda }=\sum_{X}L_{X}^{\lambda }\left[X\right]$ are still conserved. These conservation laws are said to be *unbroken*. The other conservation laws correspond to quantities $L^{\lambda }=\sum_{X}L_{X}^{\lambda }\left[X\right]+\sum_{Y}L_{Y}^{\lambda }\left[Y\right]$ that are not conserved anymore and are, therefore, named *broken* conservation laws.

The 2 (linearly-independent) right-null eigenvectors of $S^{X}$ (*i.e.*, the (sub)stoichiometric matrix for the internal species),

$$\begin{array}{cccccccccccccc} & & \text{ANT} & \text{FI} & \text{OX} & \text{CS} & \text{ACO} & \text{IDH} & \text{KGDH} & \text{SL} & \text{SDH} & \text{FH} & \text{MDH} & \\ c_{r1}= & ( & 1 & \frac{10}{11} & \frac{3}{11} & \frac{1}{11} & \frac{1}{11} & \frac{1}{11} & \frac{1}{11} & \frac{1}{11} & \frac{1}{11} & \frac{1}{11} & \frac{1}{11} & ) \end{array}\text{,}$$

and

$$\begin{array}{cccccccccccccc} & & \text{ANT} & \text{FI} & \text{OX} & \text{CS} & \text{ACO} & \text{IDH} & \text{KGDH} & \text{SL} & \text{SDH} & \text{FH} & \text{MDH} & \\ c_{r2}= & ( & 0 & -\frac{1}{33} & \frac{1}{11} & \frac{1}{33} & \frac{1}{33} & \frac{1}{33} & \frac{1}{33} & \frac{1}{33} & \frac{1}{33} & \frac{1}{33} & \frac{1}{33} & ) \end{array}\text{,}$$

are named *emergent cycles*. They define sequences of reactions that overall leave the abundances of the internal species unchanged, since $S^{X}c_{r1}=0$ and $S^{X}c_{r2}=0$ by definition, while interconverting the exchanged species as they undergo the effective reactions

(Equation 35) $${\text{ADP}}_{c}+{\text{Pi}}_{m}+\frac{3}{22}\;O_{2}+\frac{1}{11}\;\text{AcCoA}+\frac{1}{11}\;\text{CoQ}\overset{r1}{⇌}{\text{ATP}}_{c}+H_{2}O_{m}+\frac{1}{11}\;\text{CoA}+\frac{2}{11}\;{\text{CO}}_{2}+\frac{1}{11}\;{\text{CoQH}}_{2}\text{,}$$

(Equation 36) $$H_{m}^{+}+\frac{1}{22}\;O_{2}+\frac{1}{33}\;\text{AcCoA}+\frac{1}{33}\;\text{CoQ}\overset{r2}{⇌}H_{c}^{+}+\frac{1}{33}\;\text{CoA}+\frac{2}{33}\;{\text{CO}}_{2}+\frac{1}{33}\;{\text{CoQH}}_{2}\text{,}$$

whose stoichiometry is defined by $S^{Y}c_{r1}$ and $S^{Y}c_{r2}$, respectively, with $S^{Y}$ the (sub)stoichiometric matrix for the exchanged species.

##### Potential and force species

The exchanged species {Y} can be classified as either *potential* species $\left\{Y_{p}\right\}$ or as *force* species $\left\{Y_{f}\right\}$. The potential species are the largest subset of exchanged species such that the submatrix $L_{Y_{p}}^{b}$ of L for the broken conservation laws and the potential species can be inverted, *i.e.*, ${\left(L_{Y_{p}}^{b}\right)}^{-1}$ exists. The force species are the remaining species $\left\{Y_{f}\right\}=\left\{Y\right\}\setminus \left\{Y_{p}\right\}$. As discussed in other references,^38^^,^^41^ this partitioning is not unique, but different choices do not change the conclusions of the thermodynamic analysis.

In our model, we choose

$$Y_{p}\in \left\{{\text{ADP}}_{c},{\text{Pi}}_{m},H_{m}^{+},O_{2},H_{2}O_{m},\text{AcCoA},\text{CoA},{\text{CO}}_{2},\text{CoQ},{\text{CoQH}}_{2}\right\}$$

and

$$Y_{f}\in \left\{{\text{ATP}}_{c},H_{c}^{+}\right\}\text{.}$$

In Equation 33, the horizontal lines split S into(Equation 37)In Equation 34 the horizontal and vertical lines split L into(Equation 38)with *u* and *b* for unbroken and broken conservation laws, respectively.

Note that the existence of ${\left(L_{Y_{p}}^{b}\right)}^{-1}$ defines a representation of the broken conservation laws given by ${\left(L_{Y_{p}}^{b}\right)}^{-1}L^{b}$ where every broken conservation law involves only one potential species. This physically means that each quantity $L^{\lambda }$ corresponding to a broken conservation law stops being conserved once a specific potential species is exchanged. Furthermore, no new quantities $L^{\lambda }$ stop being conserved when the force species are exchanged. For this reason, each conservation law λ in Equation 34 can always be labeled using the chemical symbol of the potential species that, once exchanged, would make $L^{\lambda }$ a nonconserved quantity.

##### Nonconservative work

The general expression of the nonconservative work rate is given by

(Equation 39) $${\dot{w}}_{\text{nc}}=F_{Y_{f}}\cdot I^{Y_{f}}$$

where

(Equation 40) $$F_{Y_{f}}={\left(\mu_{Y_{f}}\cdot I-\mu_{Y_{p}}\cdot {\left(L_{Y_{p}}^{b}\right)}^{-1}L_{Y_{f}}^{b}\right)}^{⊤}$$

is the vector of the nonconservative forces, $I^{Y_{f}}$ is the vector collecting the exchange currents of the force species, and $\mu_{Y_{f}}$ (resp. $\mu_{Y_{p}}$) is the vector of the chemical/electrochemical potential of the force (resp. potential) species.

In our model, there are only two force species, namely, ${\text{ATP}}_{c}$ and $H_{c}^{+}$. The two corresponding nonconservative forces are given by

(Equation 41) $$F_{\text{ATPc}}=-\frac{\mu_{\text{AcCoa}}}{11}-\mu_{{\text{ADP}}_{c}}+\mu_{{\text{ATP}}_{c}}+\frac{2\;\mu_{{\text{CO}}_{2}}}{11}+\frac{\mu_{\text{CoA}}}{11}-\frac{\mu_{\text{CoQ}}}{11}+\frac{\mu_{{\text{CoQH}}_{2}}}{11}+\mu_{H_{2}O_{m}}-\frac{3\;\mu_{O_{2}}}{22}-\mu_{\text{Pim}}$$

and

(Equation 42) $$F_{H_{c}^{+}}=-\frac{\mu_{\text{AcCoa}}}{33}+\frac{2\;\mu_{{\text{CO}}_{2}}}{33}+\frac{\mu_{\text{CoA}}}{33}-\frac{\mu_{\text{CoQ}}}{33}+\frac{\mu_{{\text{CoQH}}_{2}}}{33}+\mu_{H_{c}}-\mu_{H_{m}}-\frac{\mu_{O_{2}}}{22}$$

while the exchange currents are

(Equation 43) $$I^{{\text{ATP}}_{c}}=\frac{1}{\delta }\left(-J_{\text{Hyd}}-\frac{1}{2}J_{\text{SERCA}}\right)\text{,}$$

and

(Equation 44) $$I^{H_{c}^{+}}=3\;J_{F1}-10\;J_{\text{OX}}\;\text{.}$$

Equation 43 is obtained by writing the rate equation for ${\text{ATP}}_{c}$ (Equation 9) according to Equation 31b: $\frac{1}{\delta }d_{t}{\left[\text{ATP}\right]}_{c}=J_{\text{ANT}}+I^{{\text{ATP}}_{c}}$. Equation 44 is obtained by recognising that $H_{c}^{+}$ is a controlled species, *i.e.*, $d_{t}{\left[H^{+}\right]}_{c}=0$, and using again Equation 31b, we can write $\frac{1}{\delta }d_{t}{\left[H^{+}\right]}_{c}=0=10\;J_{\text{OX}}-3\;J_{F1}+I^{H_{c}^{+}}$.

Notice that the nonconservative forces in Equations 41 and 42 correspond exactly to $\Delta_{r}G_{r1}^{\prime }$ and $\Delta_{r}G_{r2}^{\prime }$ of the effective reactions in Equations 35 and 36. They can therefore be rewritten as

(Equation 45) $$F_{\text{ATPc}}=\Delta_{r}G_{\text{ANT}}^{\prime }+\frac{10\;\Delta_{r}G_{F1}^{\prime }}{11}+\frac{3\;\Delta_{r}G_{\text{OX}}^{\prime }}{11}+\frac{\Delta_{r}G_{\text{TCA}}^{\prime }}{11}=\Delta_{r}G_{r1}^{\prime }$$

and

(Equation 46) $$F_{H_{c}^{+}}=-\frac{\Delta_{r}G_{F1}^{\prime }}{33}+\frac{\Delta_{r}G_{\text{OX}}^{\prime }}{11}+\frac{\Delta_{r}G_{\text{TCA}}^{\prime }}{33}=\Delta_{r}G_{r2}^{\prime }$$

where $\Delta_{r}G_{\text{TCA}}^{\prime }:=\Delta_{r}G_{\text{CS}}^{\prime }+\Delta_{r}G_{\text{ACO}}^{\prime }+\Delta_{r}G_{\text{IDH}}^{\prime }+\Delta_{r}G_{\text{KGDH}}^{\prime }+\Delta_{r}G_{\text{SL}}^{\prime }+\Delta_{r}G_{\text{SDH}}^{\prime }+\Delta_{r}G_{\text{FH}}^{\prime }+\Delta_{r}G_{\text{MDH}}^{\prime }$. We numerically compute the nonconservative forces using the latter expressions. The expression of the nonconservative work rate for our model then becomes

(Equation 47) $${\dot{w}}_{\text{nc}}=\Delta_{r}G_{r1}^{\prime }I^{{\text{ATP}}_{c}}+\Delta_{r}G_{r2}^{\prime }I^{H_{c}^{+}}\;\text{.}$$

##### Driving work

The driving work rate is in general given by the sum of two contributions,

(Equation 48) $${\dot{w}}_{\text{driv}}={\dot{w}}_{\text{driv}}^{\text{ch}}+{\dot{w}}_{\text{driv}}^{\text{in}}\text{,}$$

namely, the *chemical driving work rate*

(Equation 49) $${\dot{w}}_{\text{driv}}^{\text{ch}}=-\left(d_{t}\mu_{Y_{p}}\right)\cdot {\left(L_{Y_{p}}^{b}\right)}^{-1}L^{b}\left[Z\right]\text{,}$$

and the *interaction driving work rate*

(Equation 50) $${\dot{w}}_{\text{driv}}^{\text{in}}=\nabla_{\left[e\right]}G^{\text{in}}\left(\left[Z\right],\left[e\right]\right)\cdot d_{t}\left[e\right]\text{,}$$

Here, [Z]=([X],[Y]) is a vector collecting the concentrations of both internal and exchanged species, [e] is a vector collecting the concentrations of other interacting species (*i.e.*, ${\text{Na}}_{m}^{+}$, ${\text{Ca}}_{m}^{2+}$) that are not interconverted by the internal reactions and that do not appear in S, $\nabla_{\left[e\right]}$ is the gradient with respect to [e] and $G^{\text{in}}\left(\left[Z\right],\left[e\right]\right)$ is the interaction Gibbs free energy, whose exact expression depends on the model used to describe interactions.^41^

In our model, we compute the driving work rate over one period $t_{p}$ in the oscillatory regime (as it vanishes at steady state). The specific expression of the average chemical driving work rate is given by

$$\frac{1}{t_{p}}\int_{0}^{t_{p}}dt\;{\dot{w}}_{\text{driv}}^{\text{ch}}=\frac{1}{t_{p}}\int_{0}^{t_{p}}dt\;(-d_{t}\mu_{{\text{ADP}}_{c}}L^{{\text{ADP}}_{c}}-d_{t}\mu_{{\text{Pi}}_{m}}L^{{\text{Pi}}_{m}}-d_{t}\mu_{H_{m}^{+}}L^{H_{m}^{+}}-d_{t}\mu_{O_{2}}L^{O_{2}}/22-d_{t}\mu_{H_{2}O}L^{H_{2}O}$$

(Equation 51) $$-d_{t}\mu_{\text{AcCoA}}L^{\text{AcCoA}}/33-d_{t}\mu_{\text{CoA}}L^{\text{CoA}}/33-d_{t}\mu_{{\text{CO}}_{2}}L^{{\text{CO}}_{2}}/33-d_{t}\mu_{{\text{CoQH}}_{2}}L^{{\text{CoQH}}_{2}}/33-d_{t}\mu_{\text{CoQ}}L^{\text{CoQ}}/33)\text{.}$$

Notice that the terms corresponding to uncharged controlled species (*i.e.*, Pi_m_, O_2_, H_2_O_m_, AcCoA, CoA, CO_2_, CoQH_2_, CoQ) vanish since their chemical potential is constant over time. For the charged controlled species $H_{m}^{+}$, the quantity $L^{H_{m}^{+}}$ is still conserved since ${\left[H^{+}\right]}_{m}$ and ${\left[H^{+}\right]}_{c}$ are constant. Hence, $\frac{1}{t_{p}}\int_{0}^{t_{p}}dt\;\left(d_{t}\mu_{H_{m}^{+}}L^{H_{m}^{+}}\right)=\frac{L^{H_{m}^{+}}}{t_{p}}\int_{0}^{t_{p}}dt\;\left(d_{t}\mu_{H_{m}^{+}}\right)=0$ since the electrochemical potential is a state function. Similarly, $L^{{\text{ADP}}_{c}}={\left[\text{ADP}\right]}_{c}+{\left[\text{ATP}\right]}_{c}$ is still conserved in the open system implying $\frac{1}{t_{p}}\int_{0}^{t_{p}}dt\;\left(-d_{t}\mu_{{\text{ADP}}_{c}}L^{{\text{ADP}}_{c}}\right)=\frac{L^{{\text{ADP}}_{c}}}{t_{p}}\int_{0}^{t_{p}}dt\;\left(-d_{t}\mu_{{\text{ADP}}_{c}}\right)=0$. In conclusion, the chemical driving work rate over one period vanishes:

(Equation 52) $$\frac{1}{t_{p}}\int_{0}^{t_{p}}dt\;{\dot{w}}_{\text{driv}}^{\text{ch}}=0\;\text{.}$$

We cannot determine the explicit expression of the interacting driving work rate since our model does not provide the interaction Gibbs free energy $G^{\text{in}}\left(\left[Z\right],\left[e\right]\right)$. Thus, we compute the driving work over one period by calculating the total entropy production rate as the sum of the individual EPR of each internal reaction $\kappa_{i}$ = {ANT, F1, OX, CS, ACO, IDH, KGDH, SL, SDH, FH, MDH},

(Equation 53) $$T\dot{\sigma }=-\sum_{\kappa_{i}}\Delta_{r}G_{\kappa_{i}}^{\prime }J_{\kappa_{i}}$$

from which we subtract the nonconservative work over one period:

(Equation 54) $$\frac{1}{t_{p}}\int_{0}^{t_{p}}dt\;{\dot{w}}_{\text{driv}}=\frac{1}{t_{p}}\int_{0}^{t_{p}}dt\;\left(T\dot{\sigma }-{\dot{w}}_{\text{nc}}\right)\;\text{.}$$

##### Thermodynamic efficiency

In subsection 2, we determined the specific expressions of work rates in the second law of thermodynamics (Equation 32), accounting for the free energy exchanges between mitochondria and their surroundings that balance dissipation and maintain the mitochondrial metabolism out of equilibrium. The thermodynamic efficiency of mitochondrial metabolism is defined by identifying which of these terms play the role of free energy input and output. To do so, we further split the nonconservative work rate (Equation 47) by recognizing that the effective reaction (Equation 35) is given by the sum of 2 (mass balanced) reactions

(Equation 55) $${\text{ADP}}_{c}+{\text{Pi}}_{m}\overset{r1_{\text{out}}}{⇌}{\text{ATP}}_{c}+H_{2}O_{m}\text{,}$$

(Equation 56) $$\frac{3}{22}\;O_{2}+\frac{1}{11}\;\text{AcCoA}+\frac{1}{11}\;\text{CoQ}\overset{r1_{\text{in}}}{⇌}\frac{1}{11}\;\text{CoA}+\frac{2}{11}\;{\text{CO}}_{2}+\frac{1}{11}\;{\text{CoQH}}_{2}\;\text{.}$$

The effective reaction 55 corresponds to the net production (output) of free energy (ATP_c_) by mitochondrial metabolism, while the effective reactions 56 and 36 represent a free energy input generated by the TCA cycle, the electron transport chain and the F1F0-ATPase, respectively in its ATP synthase or hydrolyzing mode. The latter mode optimizes the proton driving force while the the first maximizes ATP synthesis. This allows us to write the nonconservative work rate (Equation 47) as

(Equation 57) $${\dot{w}}_{\text{nc}}=\Delta_{r}G_{r1_{\text{in}}}^{\prime }I^{{\text{ATP}}_{c}}+\Delta_{r}G_{r1_{\text{out}}}^{\prime }I^{{\text{ATP}}_{c}}+\Delta_{r}G_{r2}^{\prime }I^{H_{c}^{+}}\;\text{.}$$

The thermodynamic efficiency at steady state is thus given by

(Equation 58) $$\eta =-\frac{\Delta_{r}G_{r1_{\text{out}}}^{\prime }I^{{\text{ATP}}_{c}}}{\Delta_{r}G_{r1_{\text{in}}}^{\prime }I^{{\text{ATP}}_{c}}+\Delta_{r}G_{r2}^{\prime }I^{H_{c}^{+}}}\;\text{.}$$

In the oscillatory regime we also have to account for the free energy provided by the driving work and, therefore, the thermodynamic efficiency averaged over a period is

(Equation 59) $$\eta_{t_{p}}=-\frac{\int_{0}^{t_{p}}dt\;\Delta_{r}G_{r1_{\text{out}}}^{\prime }I^{{\text{ATP}}_{c}}}{\int_{0}^{t_{p}}dt\;\left(\Delta_{r}G_{r1_{\text{in}}}^{\prime }I^{{\text{ATP}}_{c}}+\Delta_{r}G_{r2}^{\prime }I^{H_{c}^{+}}+{\dot{w}}_{\text{driv}}\right)}\;\text{.}$$

In both cases, the thermodynamic efficiency quantify the amount of energy released by the synthesis of ATP normalized by the amount of energy injected in the mitochondria. In the main text, we use the notation $\bar{\eta }$ to refer to the average efficiency at steady-state or in the oscillatory regime.

##### Sensitivity analysis

We investigated the robustness of the rescuing effect of Ca^2+^ on the thermodynamic efficiency of mitochondrial metabolism. First, we added perturbations to all the leading constants ($V_{\max}$, $k_{f}$, ρ) of the model. Random perturbations in the range of [-10, 10]% and selected from a uniform distribution are applied to each parameter of the model (Table 6). In Figures S6A and S6B, we show the resulting bifurcation diagrams of mitochondrial efficiency in dependence on $\left[{\text{IP}}_{3}\right]$ (for $\left[\text{AcCoA}\right]=10\;\mathrm{\mu M}$) and [AcCoA] (for $\left[{\text{IP}}_{3}\right]=0.20\;\mathrm{\mu M}$). For each realization, the thermodynamic consistency criterion is satisfied, *i.e.* all Gibbs free energies of reactions are negative. In all simulations, an increase in efficiency at the onset of Ca^2+^ oscillations is visible, which supports the robustness of the results against cellular variability.

We also performed a complementary sensitivity analysis with a larger perturbation range ([-75,75]%) to assess the impact of variability of one leading constant at a time on steady-state regime ($\left[{\text{IP}}_{3}\right]=0.20\;\mathrm{\mu M}$) and oscillatory regimes ($\left[{\text{IP}}_{3}\right]=0.50\;\mathrm{\mu M}$) with $\left[\text{AcCoA}\right]=10\;\mathrm{\mu M}$. The fold-change to reference conditions (Table 6) in key variables and the absolute change period are shown in Figures S6C and S6D, respectively.

Cellular respiration becomes endergonic ($\Delta G_{\text{Ox}}>0$) upon moderate to large variations in $k_{\text{Hyd}}$, $\rho_{F1}$ (decrease by 20% or more) and $\rho_{\text{res}}$ (increase by 20% or more) or upon large variations in $V_{\max}^{\text{SERCA}}$, $V_{\max}^{\text{ANT}}$ (decrease by 50% or more) and $V_{\max}^{\text{IDH}}$ (increase by 50% or more). The reaction catalyzed by fumarate hydratase also becomes endergonic ($\Delta G_{\text{FH}}>0$ – data not shown) for moderate to large perturbations of $\rho_{\text{res}}$ (decrease by 20% or more) and large perturbations of $V_{\max}^{\text{CS}}$ (decrease by 75%) or $\rho_{F1}$ (increase by 50% or more). Those perturbations indeed lead to an accumulation of malate, which affects the spontaneity of the reaction. We exclude these thermodynamically unfavorable conditions from the remaining of the discussion. The efficiency is mainly impacted by the two parameters related to OXPHOS ($\rho_{\text{res}}$ and $\rho_{F1}$), SERCA-independent ATPc hydrolysis ($k_{\text{Hyd}}$), by Ca^2+^ exchanges ($V^{\text{LEAK}}$, $V_{\max}^{{\text{IP}}_{3}R}$, $V_{\max}^{\text{UNI}}$, $V_{\max}^{\text{NCX}}$ and, to a lesser extent, $V_{\max}^{\text{SERCA}}$), nucleotide exchanges ($V_{\max}^{\text{ANT}}$) and some enzymes of the TCA cycle ($V_{\max}^{\text{CS}}$ and $V_{\max}^{\text{IDH}}$). Generally, Ca^2+^ transfer to mitochondria enhances the efficiency while Ca^2+^ release in the cytosol decreases the steady-state efficiency. Large perturbations in $V_{\max}^{{\text{IP}}_{3}R}$ (+75%) or in $V_{\max}^{\text{IDH}}$ (-75%) induce oscillations, respectively via stimulation of GPCRs and by limitation of SERCA pumps by ATP_c_ deficiency, respectively. Ca^2+^ oscillations have a beneficial impact on the efficiency in the first case because the increased production of NADH by activated mitochondrial dehydrogenases results in an increased production of ATP_c_. In the second case, the global rate of the TCA cycle is limited by the rate of isocitrate dehydrogenase, which leads to reduced ΔΨ, efficiency and ATP_c_ level.

Similar observations hold in the oscillatory regime. $\Delta G_{\text{Ox}}$ is even more susceptible to become positive upon changes in $k_{\text{Hyd}}$, $\rho_{F1}$, $\rho_{\text{res}}$, $V_{\max}^{\text{ANT}}$, and $V_{\max}^{\text{IDH}}$, and variations in $V_{\max}^{\text{NCX}}$ (decrease by 50% or more) and $V_{\max}^{\text{CS}}$ (increase by 50% or more) can also make cellular respiration endergonic, probably because increasing ΔΨ affects the spontaneity of the reaction. Note that decreasing $V_{\max}^{{\text{IP}}_{3}R}$ (by 75%) leads to the disappearance of oscillations and the associated efficiency is lower than for the oscillatory regimes generated by larger values of that parameter. The kinetics of mitochondrial exchange processes ($V_{\max}^{\text{NCX}}$ and $V_{\max}^{\text{UNI}}$) have a more pronounced impact on the efficiency of oscillatory regimes as compared to steady-state efficiency, with a beneficial impact of mitochondrial Ca^2+^ accumulation (low $V_{\max}^{\text{NCX}}$ and high $V_{\max}^{\text{UNI}}$) on the efficiency.

Overall, the robustness of the rescuing effect of Ca^2+^ oscillations on the thermodynamic efficiency of mitochondria is supported by the sensitivity analysis.
