# Supplementary material for: Calcium oscillations optimize the energetic efficiency of mitochondrial metabolism
Source: iScience. 2024 Feb 1;27(3):109078. doi: 10.1016/j.isci.2024.109078 (PMC10875125; doi:10.1016/j.isci.2024.109078)
Supplement: Document S1. Figures S1–S6 [file mmc1.pdf]

iScience, Volume *27*

Supplemental information

Calcium oscillations optimize the energetic efficiency of mitochondrial metabolism

Valérie Voorsluijs, Francesco Avanzini, Gianmaria Falasco, Massimiliano Esposito, and Alexander Skupin

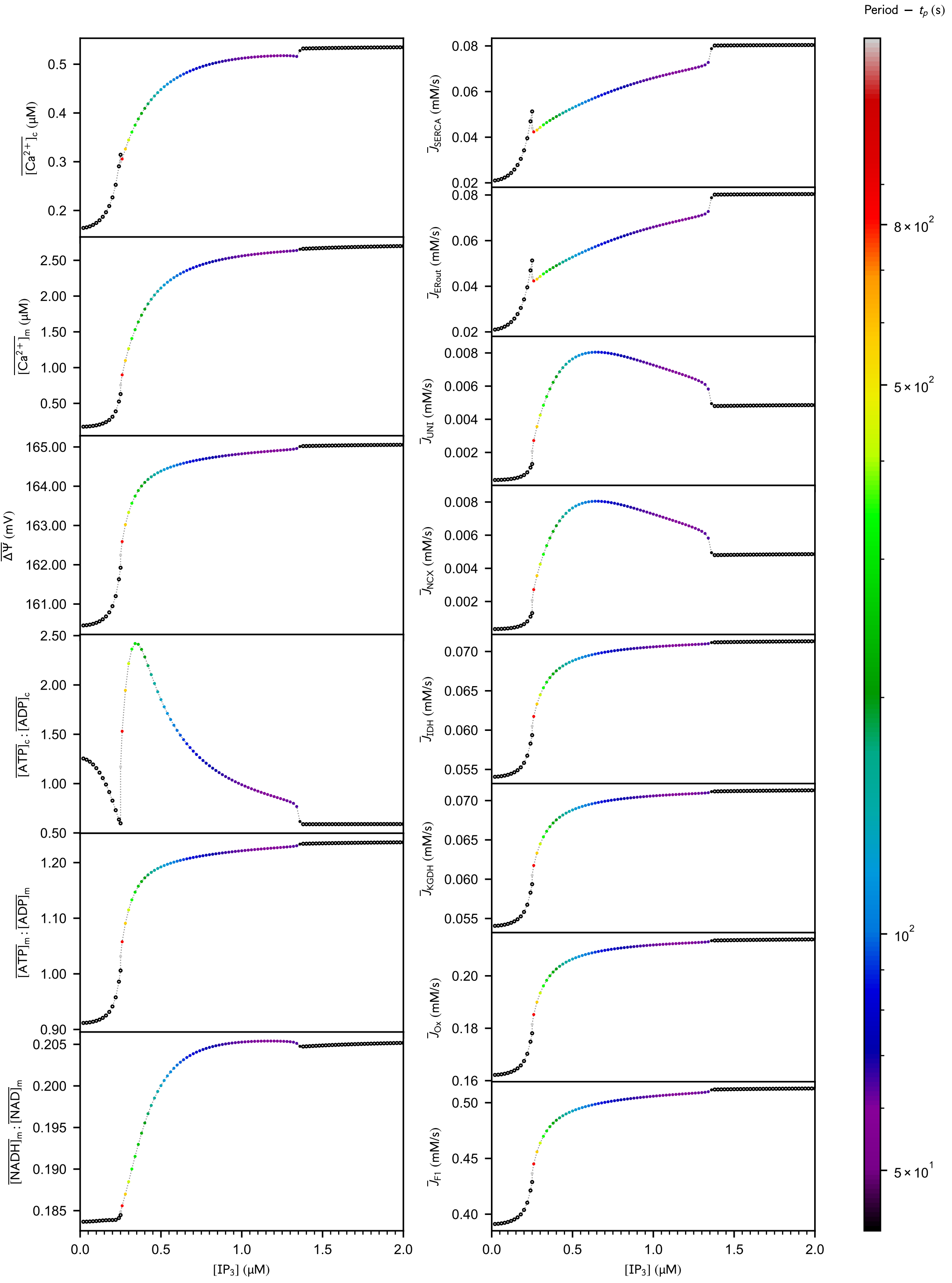

FIGURE S1: Average values of key variables and fluxes *vs.* [IP$_3$] as complementary figures to the bifurcation diagrams shown in Fig. 2*D* and Fig. 3*B* bottom and portrait phases in Fig. 4*C*. Note that $\bar{J}_{\text{IDH}}$ and $\bar{J}_{\text{KGDH}}$ are indistinguishable. Empty and filled dots correspond to steady-state or period-averaged quantities, respectively. Parameter values are the same as in Fig. 2*D*.

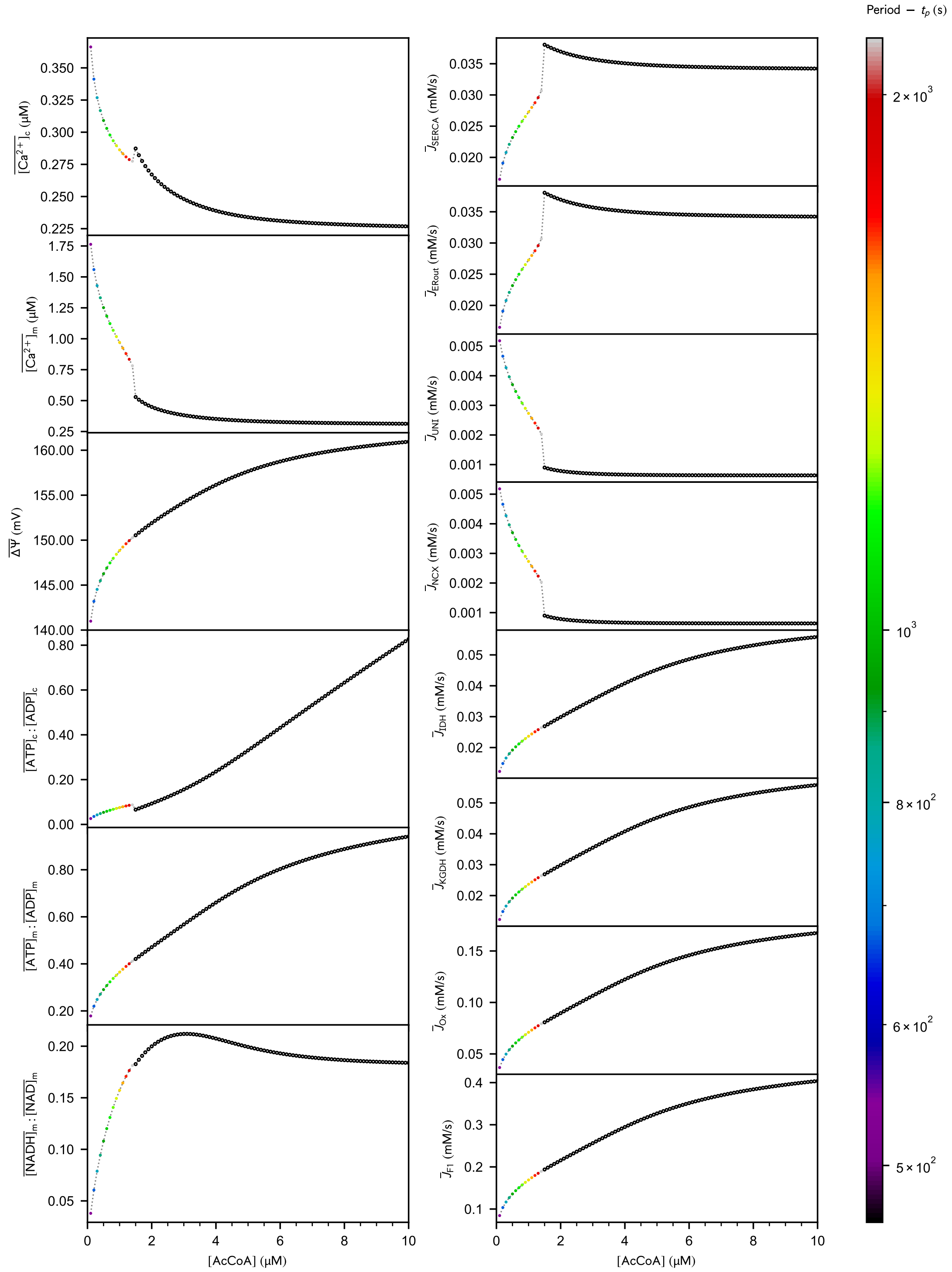

FIGURE S2: Average values of key variables and fluxes *vs.* [AcCoA] as complementary figures to the bifurcation diagrams shown in Fig. 2*E* and Fig. S3*B* bottom. Note that $\bar{J}_{\mathrm{IDH}}$ and $\bar{J}_{\mathrm{KGDH}}$ are indistinguishable. Empty and filled dots correspond to steady-state or period-averaged quantities, respectively. Parameter values are the same as in Fig. 2*E*.

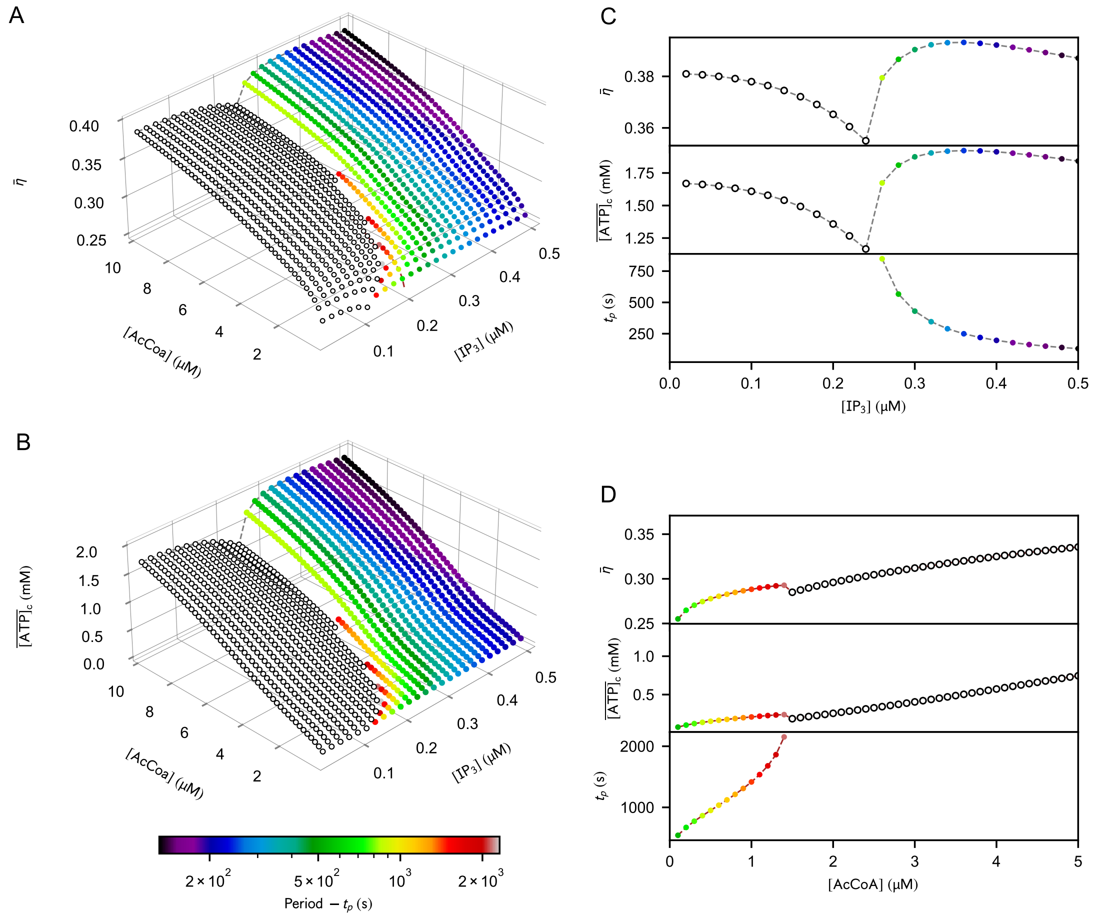

Figure S3: Efficiency of mitochondrial metabolism (η), average cytosolic ATP concentration ($\overline{[ATP]}_c$ (mM)) and period of Ca^{2+} oscillations (t_p) as functions of [AcCoA] and [IP_3]. (*A-B*) Summary 3D plots. IP_3 plays a dominant role in the transition between steady-state and oscillations, which usually takes place for concentrations of IP_3 between 0.2 and 0.3 μM. The onset of oscillations can be triggered for smaller [IP_3] in the presence of a very low level of AcCoA (e.g. [AcCoA] $\leq$ 1 μM), which supports the role of Ca^{2+} oscillations as a rescuing mechanism aiming to improve the efficiency of energy production in stressing situations such as substrate-limited. For more clarity, representative behaviors of the efficiency (top panels), $\overline{[ATP]}_c$ (mM) (middle panels) and period (low panels) were plot for (grey lines - *C*) [AcCoA] = 10 μM and (brown lines - *D*) [IP_3] = 0.20 μM, as complementary figures to the bifurcation diagrams shown in Fig. 2*D* and 2*E*, respectively. Note that maxima in efficiency and in $\overline{[ATP]}_c$ (mM) can also be observed when [AcCoA] is varied. Increments in concentrations are of 0.2 μM (*A-B*) or 0.1 μM (*D*) for AcCoA and of 0.2 μM (*A* – *C*) for IP_3.

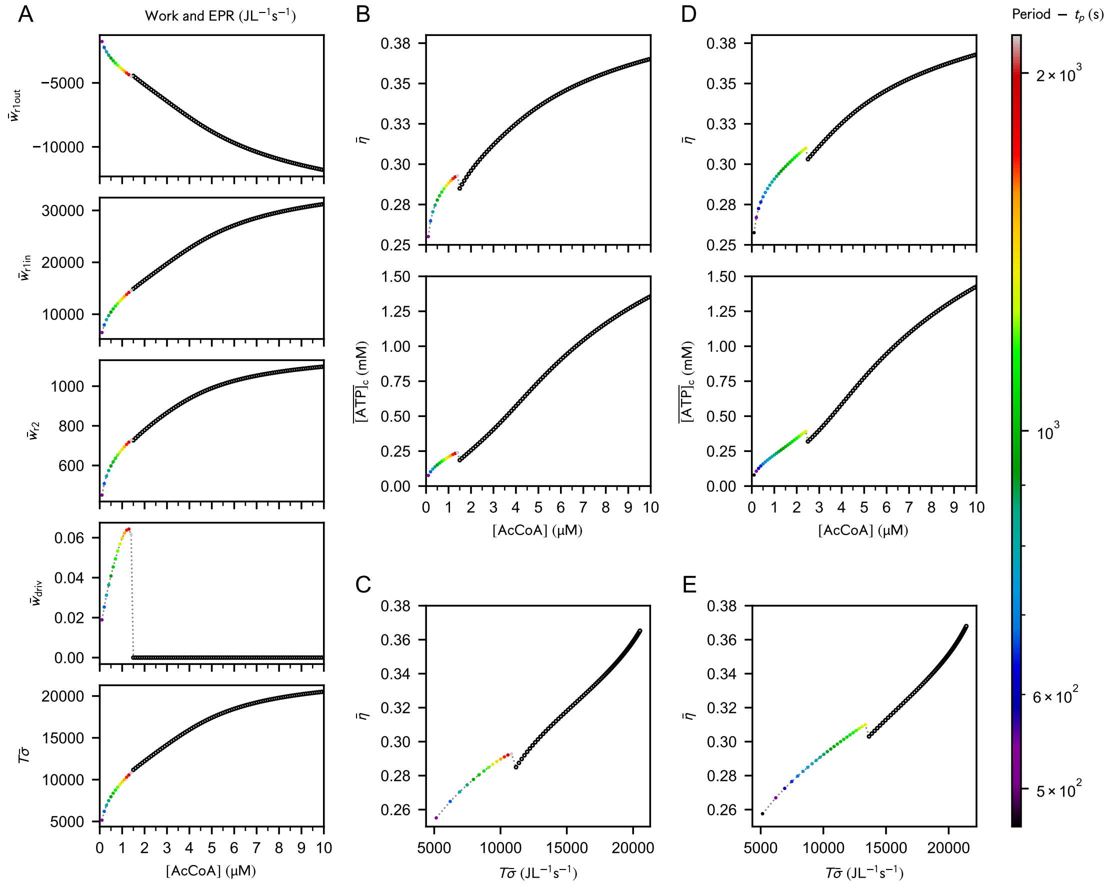

Figure S4: Stimulation of mitochondrial metabolism by AcCoA impacts Ca^{2+} dynamics *via* the Ca^{2+}-metabolism cross-talk. (*A*) Nonconservative work contributions, driving work and dissipation for different [AcCoA]. The driving work represents less than 0.0006% of the EPR. At high stimulation, oscillations disappear in the favor of a nonequilibrium steady-state regime. (*B*) Efficiency and ATP_c concentration as a function of [IP_3]. (*C)* Efficiency as a function of the total dissipation for the same range of [AcCoA] as in (*A*) and (*B*). (*D-E*) Plots corresponding to (*B-C*) for $V_{max}^{SERCA} = 0.096\,\mu M\,s^{-1}$. Empty and filled dots correspond to steady-state or period-averaged quantities, respectively. Unless specified otherwise, parameter values are the same as in Fig. 2*E*.

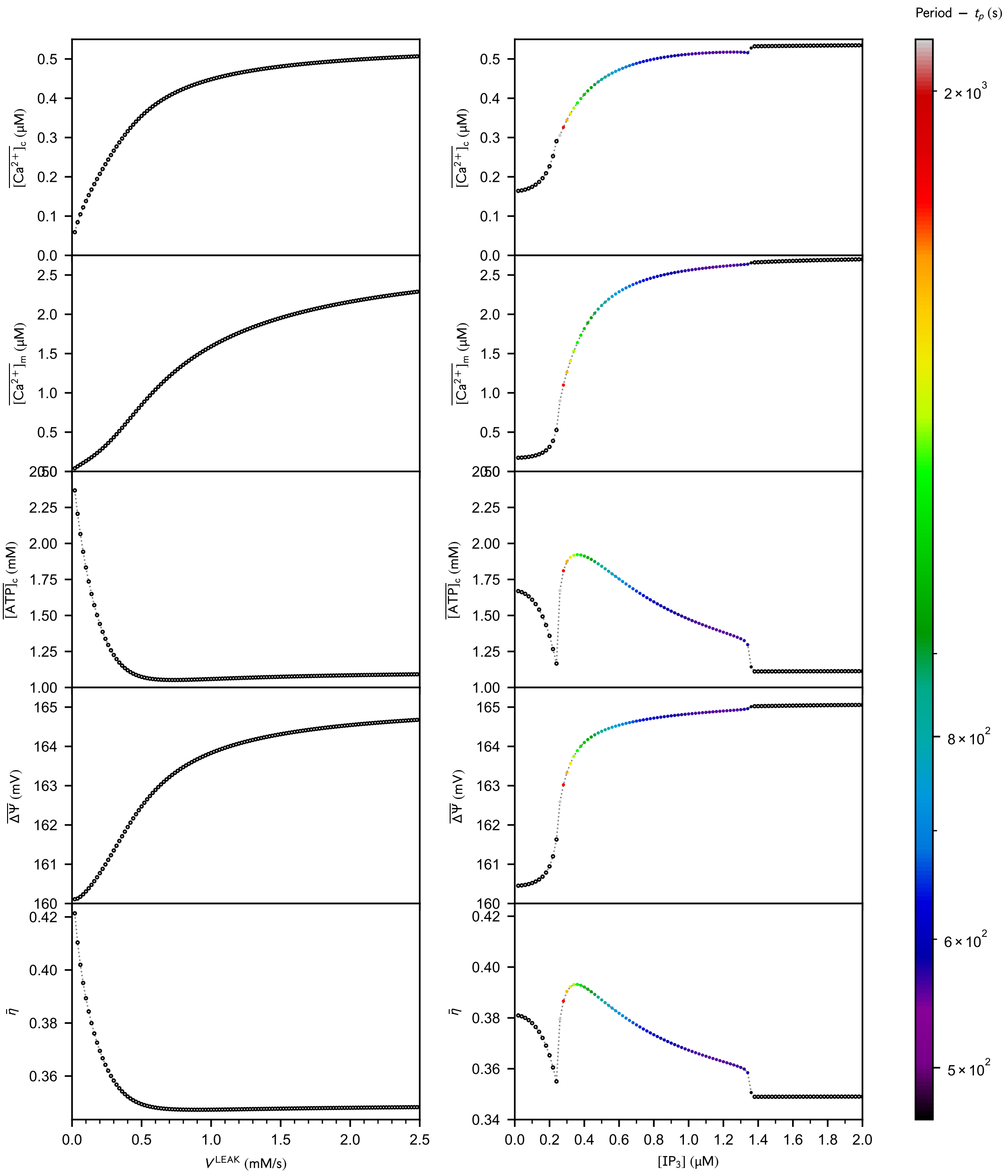

FIGURE S5: Average values of key variables *vs.* V^{LEAK} for $[\text{IP}_3] = 0.10\ \mu$M. The corresponding plots *vs.* $[\text{IP}_3]$ for $V^{\text{LEAK}} = 0.15$ mM s^{-1} (*cf.* Table 4) are shown on the right for comparison. Empty and filled dots correspond to steady-state or period-averaged quantities, respectively. The other parameter values are the same as in Fig. 2*D*.

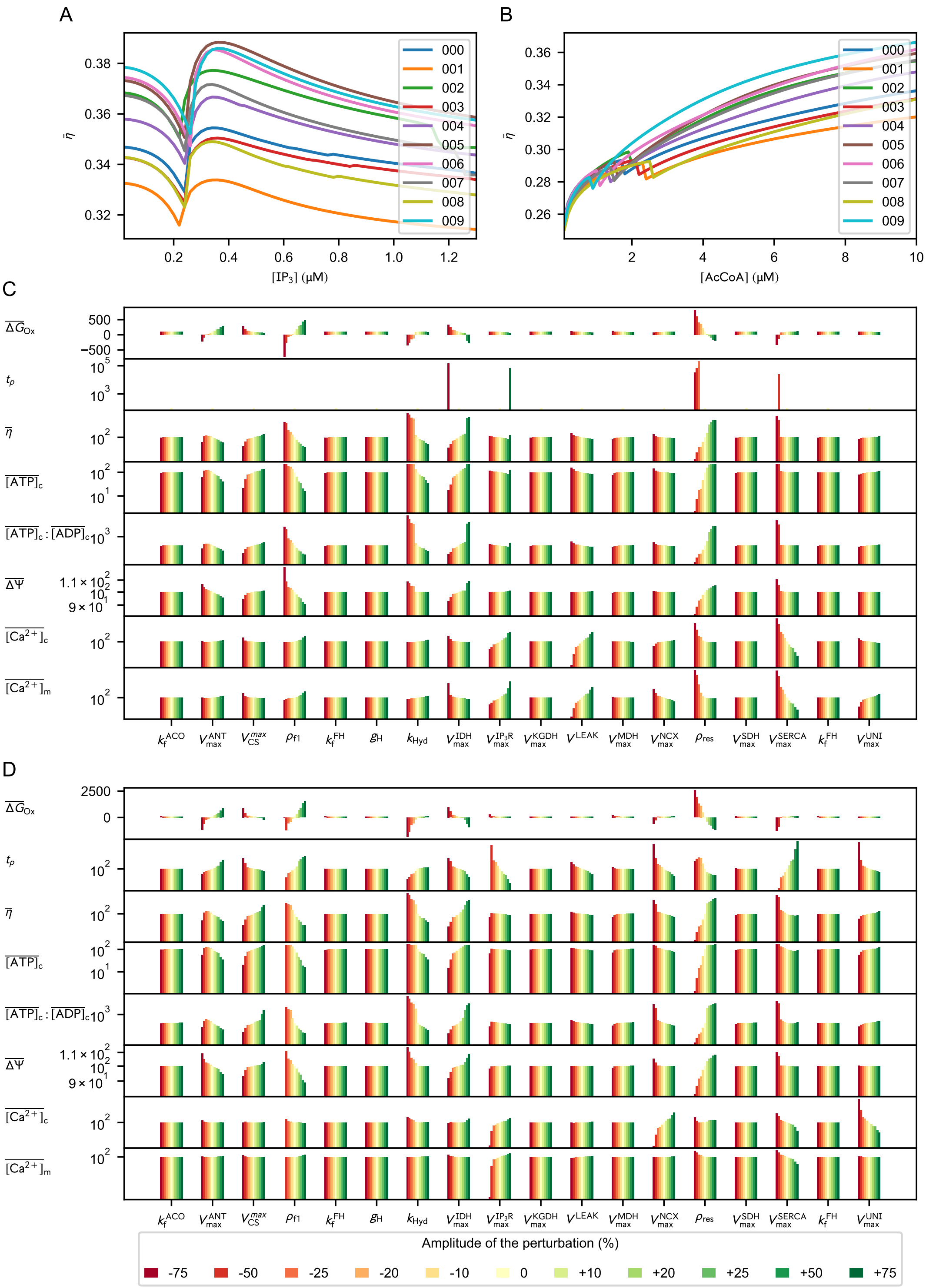

FIGURE S6: Sensitivity analysis. (A – B) Bifurcation diagrams for [AcCoA] = 10 (μM) and [IP_3] = 0.2 (μM). The values of the leading constants can be found in Table 6. (C – D) Fold-change in Gibbs free energy of cellular respiration, thermodynamic efficiency, cytosolic ATP concentration, ATP_c:ADP_c ratio, mitochondrial membrane potential, cytosolic Ca^{2+} concentration, mitochondrial Ca^{2+} concentration, and (change in) absolute period of Ca^{2+} oscillations. Oscillations can be induced by perturbations of the reference steady-state and *vice-versa*. If oscillations are present, the calculations are based on the period-averaged value of the quantity. For the period bar plot, the absence of a bar indicates a steady-state regime.
